# Community context influences the conjugation efficiency of *Escherichia coli*

**DOI:** 10.1093/femsmc/xtae023

**Published:** 2024-07-27

**Authors:** Misshelle Bustamante, Floor Koopman, Jesper Martens, Jolanda K Brons, Javier DelaFuente, Thomas Hackl, Oscar P Kuipers, G Sander van Doorn, Marjon G J de Vos

**Affiliations:** GELIFES, University of Groningen, 9747 AG Groningen, The Netherlands; GELIFES, University of Groningen, 9747 AG Groningen, The Netherlands; GELIFES, University of Groningen, 9747 AG Groningen, The Netherlands; GELIFES, University of Groningen, 9747 AG Groningen, The Netherlands; Centro Nacional de Biotecnología (CNB), CSIC, 28049 Madrid, Spain; GELIFES, University of Groningen, 9747 AG Groningen, The Netherlands; GBB, University of Groningen, 9747 AG Groningen, The Netherlands; GELIFES, University of Groningen, 9747 AG Groningen, The Netherlands; GELIFES, University of Groningen, 9747 AG Groningen, The Netherlands

**Keywords:** conjugation rate, polymicrobial community, antimicrobial resistance, *E. coli*, ecology, pOXA-48

## Abstract

In urinary tract infections (UTIs), different bacteria can live in a polymicrobial community consisting of different species. It is unknown how community members affect the conjugation efficiency of uropathogenic *Escherichia coli*. We investigated the influence of individual species often coisolated from urinary infections (UTI) on the conjugation efficiency of *E. coli* isolates in artificial urine medium. Pairwise conjugation rate experiments were conducted between a donor *E. coli* strain containing the pOXA-48 plasmid and six uropathogenic *E. coli* isolates, in the presence and absence of five different species commonly coisolated in polymicrobial UTIs to elucidate their effect on the conjugation efficiency of *E. coli*. We found that the basal conjugation rates of pOXA-48, in the absence of other species, are dependent on the bacterial host genetic background. Additionally, we found that bacterial interactions have an overall positive effect on the conjugation rate of pOXA-48. Particularly, Gram-positive enterococcal species were found to enhance the conjugation rates towards uropathogenic *E. coli* isolates. We hypothesize that the nature of the coculture and physical interactions are important for these increased conjugation rates in an artificial urine medium environment.

## Teaser

Our study unveils the community impact on the conjugation efficiency of uropathogenic *E. coli* isolates, with Gram-positive species, particularly enterococci, enhancing the conjugation efficiency.

## Introduction

Antimicrobial resistance (AMR) poses a significant challenge to global public health (Murray et al. 2022). The intense use of antibiotics has led to the emergence and spread of multidrug resistance in pathogenic bacteria (Polianciuc et al. 2020). This threatens the effectiveness of antibiotics, and therefore our ability to cure infections (Prestinaci et al. 2015, Aslam et al. 2018).

Bacteria can acquire AMR by horizontal exchange of genetic material among related or unrelated bacterial species, in a process referred to as ‘horizontal gene transfer’ or HGT (Ochman et al. 2000, Hall et al. 2017). The exchange of genetic material between microbes can occur in various ways, often by a process called conjugation (Furuya and Lowy 2006). Conjugation involves the physical contact between donor and recipient cells and typically a self-transmissible or mobilizable plasmid (Ochman et al. 2000).

Conjugative or mobilizable plasmids are the most common transmission vectors for AMR genes (Boerlin and Reid-Smith 2008, Partridge et al. 2018, Ares-Arroyo et al. 2022) and the major drivers of HGT within bacterial communities (Bottery 2022). AMR genes and HGT have been observed within the human microbiome. A major hotspot for antibiotic resistance is, for instance, the gut microbiome of humans and animals (San Millan 2018), where rich dynamics of plasmid transfer have been observed (Frazão et al. 2023).

Although the ecology and functioning of microbial communities are typically studied in one specific environment at a time (Smillie et al. 2011), it is known that the rate of HGT is strongly dependent on the abiotic and the biotic environments (Sessitsch et al. 2023). For instance, resource availability and temperature (Pallares-Vega et al. 2021) or other abiotic factors such as salt stress (Beuls et al. 2012), can significantly affect the rate at which HGT via conjugation occurs. Biotic factors, such as the presence of ecological interaction partners, can affect the spread of conjugative plasmids within and between host species (Bottery 2022). Additionally, the horizontal transmission of plasmids can be limited by bacterial diversity due to the ‘dilution effect’; i.e. the phenomenon whereby living alongside less proficient host species reduces the expected infection risk for a focal host species (Kottara et al. 2021). The microbial context, such as the presence of competitors, can also determine the cost and benefits of conjugative plasmid maintenance (Sünderhauf et al. 2023). Moreover, ecological interactions can alter factors such as growth rate and population densities, which together can affect the cost of plasmid carriage and conjugation rates (Duxbury et al. 2021).

It is still an open question to what extent bacterial interactions affect the transfer of antibiotic resistance by HGT via conjugation in bacterial communities. Given that complex communities are difficult to study, we investigate this question for a simple and tractable, yet relevant, system consisting of bacterial species often coisolated from elderly patients diagnosed with urinary tract infections (UTIs) (Croxall et al. 2011b). The prevalence of AMR in such communities is high (Croxall et al. 2011b), and there has been an increase in AMR and multidrug resistance in recent years (Trautner et al. 2022).

In such communities, Gram-positive species live together with Gram-negative species (de Vos et al. 2017, Zandbergen et al. 2021), but the importance of Gram-positive species for UTIs is often overlooked. Yet, Gram-positive bacteria are an important cause of nosocomial infections (Furuno et al. 2005, Cong et al. 2019). Enterococci, for instance, have been shown to facilitate polymicrobial infections, leading to more complicated pathogenesis and poorer prognoses (Chong et al. 2017, Barshes et al. 2022), and they can compromise the efficacy of antimicrobial agents by promoting colonization, proliferation, and persistence of diverse pathogenic bacteria (Xu et al. 2023). Furthermore, they can act as reservoirs for the transmission of AMR and virulence determinants (Coburn et al. 2007, Xu et al. 2021).

Here, we investigate the effect of ecological interactions between *Escherichia coli* and other bacterial species often identified in polymicrobial UTIs on the conjugation rate of pOXA-48 towards uropathogenic *E. coli*. pOXA-48 is a plasmid with a broad host range, carrying the resistance gene *bla*_OXA-48_ that confers resistance to multiple ꞵ-lactam antibiotics (Poirel et al. 2004, 2012), including carbapenems, which are last-resort antibiotics used to treat multidrug-resistant infections (Bradley et al. 1999, Papp-Wallace et al. 2011). It is an important conjugative plasmid in the clinical setting, known for its rapid dissemination within hospital patients and has a worldwide distribution (Pitout et al. 2019, León-Sampedro et al. 2021).

Specifically, we study the effect of ecological interactions on the conjugation efficiency in uropathogenic *E. coli* isolates, by performing pairwise conjugation assays in the presence of *Enterococcus faecium, Enterococcus faecalis, Staphylococcus simulans, Pseudomonas aeruginosa*, and *Proteus mirabilis* in artificial urine medium (AUM). All isolates, except for *P. aeruginosa*, were collected from elderly patients who were diagnosed with polymicrobial UTIs (Croxall et al. 2011b). It is important to mention that the term conjugation ‘efficiency’ is used as a qualitative description of the ability of recipient *E. coli* isolates to take up pOXA-48 plasmid. On the other hand, conjugation ‘rate’ is used to refer to the quantitative assessment of the transfer of pOXA-48 plasmid to uropathogenic *E. coli*.

## Materials and methods

### Bacterial isolates

Nine *E. coli* isolates were selected from a previous study where samples were collected from elderly patients diagnosed with polymicrobial UTIs (Croxall et al. 2011b). They were selected based on their sensitivity to ampicillin. Multi locus sequence typing, followed by phylotyping (Beghain et al. 2018) of these uropathogenic *E. coli* isolates was performed previously by Croxall et al. (2011a). Initial conjugation experiments aimed at testing their ability to take up pOXA-48 plasmid resulted in six final uropathogenic *E. coli* isolates that were used as recipients in pairwise mating assays in the presence of UTI community members. Plasmid transfer in these isolates was confirmed by Polymerase Chain Reaction 'PCR' with specific primers for pOXA-48 plasmid (see the section ‘DNA extraction and PCR in Materials and methods’).

The donor strain ꞵ3914 was a diaminopimelic acid (DAP) auxotrophic *E. coli* strain, exhibiting resistance to various antibiotics, including kanamycin (Roux et al. 2007) and harbouring the pOXA-48 plasmid (Alonso-del Valle et al. 2021). This plasmid codes for the *bla*_OXA-48_ gene, which confers resistance to ꞵ-lactam antibiotics (Poirel et al. 2004), including penicillins and carbapenems (Poirel et al. 2012).

Four community members were collected from the same study as the uropathogenic *E. coli* isolates (Croxall et al. 2011b), and were also selected upon their sensitivity to ampicillin. These belonged to three Gram-positive species: *E. faecium, E. faecalis*, and *S. simulans*, and one Gram-negative species: *P. mirabilis*. Additionally, we investigate the interaction with the Gram-negative *P. aeruginosa*, because this species is frequently recovered from polymicrobial UTIs (de Vos et al. 2017). Because the polymicrobial UTI *P. aeruginosa* isolates in our collection were highly resistant to ampicillin, we used PAO1, which was less resistant to ampicillin.

### AUM

We use a modified version of AUM (Brooks and Keevil 1997, de Vos et al. 2017). It contained bacto peptone L37 1 g/l (Sigma), sodium bicarbonate 2.1 g/l (Roth), urea 7.5 g/l (Roth), sodium chloride 5.2 g/l (Sigma), sodium sulfate anhydrous 1.2 g/l, ammonium chloride 1.3 g/l (Sigma), and potassium dihydrogen phosphate 0.95 g/l added as solids; yeast extract 0.1 ml/l from 5 g/100 ml stock, lactic acid 0.1 ml/l (Roth), citric acid 0.8 ml/l from 10 g/20 ml stock, uric acid 7 ml/l from 1 g/100 ml in 1 M NaOH stock, creatinine 16 ml/l from 5 g/100 ml stock, calcium chloride dihydrate 29.60 µl/l from 1 g/10 ml stock, iron(II) sulfate heptahydrate 12 µl/l from 10 g/100 ml stock, and magnesium sulfate heptahydrate 2.45 ml/l from 1 g/10 ml stock were added as liquids.

### Efficiency of uropathogenic *E. coli* to take up pOXA-48 plasmid via conjugation in LB

This protocol was used to assess, rather qualitatively, the ability of the uropathogenic *E. coli* isolates to take up the pOXA-48 plasmid, and it was adapted from Alonso-del Valle et al. (2021, 2023). Donor ꞵ3914 and recipient *E. coli* strains were streaked on CHROMagar plates supplemented with kanamycin 30 µg/ml (Sigma) and 300 µM DAP (Sigma), for the donor; and no antibiotic for the recipients, given that they were sensitive to most antibiotics used for the treatment of UTIs. The plates were incubated overnight at 37°C. The next day, three independent colonies were picked from each isolate and grown overnight in 2 ml of Lysogeny broth (LB) at 37°C and continuous shaking at 200 rpm. Donor cultures were grown with 30 µg/ml kanamycin and 300 µM DAP.

The following day, the overnight cultures, which were at that time in stationary phase, were mixed in a 5:1 donor-to-recipient volume ratio. This relationship was established after testing several donor-to-recipient proportions, and this ratio resulted as the most effective for plasmid transfer. The experiment was performed in triplicates; 50 µl of the donor culture and 10 µl of the recipient culture were gently mixed by pipetting in 0.5 ml tubes. The full 60 µl droplets were spotted in the middle of LB agar (Sigma) plates without antibiotics but with 300 µM DAP (Sigma), and left to air-dry in the flow cabin, after which they were incubated at 37°C for 4 h to recover transconjugants. This mating time is short enough to reduce the chances of secondary conjugation events from transconjugants to recipients and the impact of potential differences in donor, recipient, and transconjugant growth rates on conjugation frequency determination (Alonso-del Valle et al. 2023). The controls consisted of 60 µl of isolated cultures of donor or recipient; each in triplicates, which were also spotted in the middle of individual LB agar (Sigma) plates with 300 µM DAP (Sigma).

After incubation, a metal loop was used to scoop out the biomass of each of the droplets, which were immediately washed and resuspended by pipetting in tubes containing 2 ml of sterile 0.9% NaCl solution. These were further diluted in serial 10-fold dilutions from 10^1^ until 10^7^ using a 96-well plate: 200 µl of resuspension was added to the first well and the remaining wells contained 180 µl of 0.9% NaCl solution. Then, 20 µl from the first well was taken and mixed with the next well. This step was repeated for all columns of the well plate. A multichannel pipette was used to transfer 10 µl from each dilution at the first quarter of a round agar plate, which was tilted 90° to let the eight droplets slide down until the end of the plate. Every donor and recipient mix, as well as the controls (donors in isolation and recipients in isolation), were plated on transconjugant-selective LB agar plates with ampicillin 100 µg/ml where only transconjugants should grow. As an additional negative control, every donor and recipient mix, as well as the controls (donors in isolation and recipients in isolation) were plated on LB agar plates with kanamycin 30 µg/ml (Sigma) without DAP, to make sure that neither donor nor recipients in isolation nor transconjugants would grow.

After overnight incubation, glycerol stocks were made from the transconjugants. Given that this was a relatively crude, qualitative method to assess conjugation, a more quantifiable method was later applied to determine pOXA-48 conjugation rates in the presence and absence of UTI community members in AUM.

### DNA extraction and PCR

To confirm plasmid transfer to the six *E. coli* isolates, DNA was extracted using a previously in-house developed ultra-fast DNA extraction method for *E.coli* described in Brons et al. (2020). Primers for amplifying the resistance gene *bla*_OXA-48_ for ꞵ-lactam antibiotics on the pOXA-48 plasmid were adopted from Poirel et al. (2004). A 20-mer forward primer, designated Oxa-48 Fw (5′-TTG GTG GCA TCG ATT ATC GG-3′) was combined with a 21-mer reverse primer, designated Oxa-48 Rev (5′-GAG CAC TTC TTT TGT GAT GGC-3′). This primer combination was tested and optimized.

PCR mixtures were prepared with the following components: 5.0 μl of 10x Roche buffer (Roche, Basel, Switzerland), 0.8 μl of 50 mM MgCl_2_ (Merck, Darmstadt, Germany), 1.0 μl of 100% dimethyl sulfoxide, 0.5 μl of 20 mg/ml bovine serum albumin (Merck), 1.0 μl of 10 mM deoxyribonucleoside triphosphate mix, 1.0 μl of 10 μM of each primer, and 0.2 μl of 5 U/μl Taq DNA Polymerase (Roche). Molecular biology-grade water (Thermo Fisher Scientific, Waltham, USA) was added to a total volume of 50 μl in a 0.2-ml microfuge tube. Finally, 1.0 μl of template DNA was added. The mixtures were incubated in a Mastercycler Nexus PCR thermal cycler (Eppendorf, Hamburg, Germany) with the following program: initial denaturation of double-stranded DNA for 5 min at 95°C; 35 cycles consisting of 1 min at 95°C, 30 s at 56°C, and 2 min at 72°C; and extension for 7 min at 72°C.

All amplification products were analyzed by electrophoresis in 1.0% (w/v) agarose gels, followed by ethidium bromide staining (1.2 mg/l ethidium bromide in 1× Tris-acetate–EDTA) (Sambrook et al. 1989, Mullis 1990), destaining (1× Tris-acetate), and visualization under UV. Amplicons of 743 bp in size were detected, and no side products were observed, confirming plasmid transfer to the six *E. coli* isolates.

### Conjugation rates of pOXA-48 to uropathogenic *E. coli* with and without community members on AUM

We performed a quantifiable method based on (León-Sampedro et al. 2021, DelaFuente et al. 2022, Alonso-del Valle et al. 2023) to determine the conjugation of pOXA-48 from donor strain ꞵ3914 to six uropathogenic *E. coli* isolates in the presence and absence of five members of the polymicrobial UTI community in AUM media. Each conjugation experiment was performed in a single day and consisted of six assays: the ‘basal conjugation rate’ assay with only donor and recipient; and the pairwise ‘community member assays’ where each of the five other species were added individually to the donor and recipient combination. Every assay was performed with three biological replicates.

A scoop from −80°C glycerol stocks was taken to grow overnight cultures of donor strain ꞵ3914, recipient *E. coli* and community members with 2 ml of 1x AUM. The donor strain was grown with 30 µg/ml kanamycin (Sigma) and 300 µM DAP (Sigma). The recipient and community member strain cultures had no additives. They were incubated for 24 h at 37°C with continuous shaking at 200 rpm.

After 24 h, optical density measurements at 600 nm (OD600) measurements were taken of all strains using a 1-ml culture in a disposable cuvette in a spectrophotometer. The population sizes were inferred from these OD600 measurements, by diluting the cultures below 0.4 OD and calculating their true OD based on the dilution factor used for each strain. Each culture was then further diluted to obtain a starting population size that, in combination, would maintain a 5:1:1 OD600 ratio between donor, recipient, and community member, respectively. These proportions were the same as used for the ‘Efficiency of uropathogenic *E. coli* to take up pOXA-48 plasmid via conjugation in LB’ protocol (see the section ‘Materials and methods’). Specifically, the OD600 values used were 1, 0.2, and 0.2, respectively; except in the case of *E. faecium* and *E. faecalis*, where the OD was always lower than 0.2.

For the assessment of the basal conjugation rates of pOXA-48 plasmid to *E. coli*, 50 µl of donor and 50 µl of recipient were added and gently mixed in a 0.5-ml tube, preserving a 5:1 OD600 ratio. This combination was the control of the experiment, the basal conjugation rate. For the assessment of the effect of other species frequently co-occurring in polymicrobial UTIs on the conjugation rates, 50 µl of donor and recipient were also added to a 0.5-ml tube with an additional volume from the individual community member culture that was dependent on the OD, but always close to 50 µl, preserving the 5:1:1 OD600 ratio of donor, recipient, and community member. If the OD of the undiluted culture was <0.2, which was always the case with *E. faecium* and *E. faecalis*, then exactly 50 µl of it was added to the tube. Therefore, the total droplet volume for the basal conjugation assay was 100 and 150 µl for the individual community member assays. For mixing, vortexing was avoided, and the tubes were gently struck several times. All droplets were spotted in the middle of 1x AUM agar plates containing DAP. These plates were prepared using 50% Micro agar (15 g/l) (Duchefa Biochemie), 50% 2x AUM and 300 μM DAP. The droplets were left to dry and incubated to allow for conjugation at 37°C for 1 h. This was performed in triplicates for every combination of donor + recipient and of donor + recipient + single other species.

After 1 h of incubation, the plates were removed from the incubator. A sterile toothpick was used to cut out the piece of agar with the droplet. Subsequently, the agar segment was crushed and resuspended in 1 ml sterile 0.9% NaCl solution. Each tube was inverted and gently shaken 30 times to wash off the cells from the agar. Further 10-fold dilutions until 10^4^ were made before plating 100 µl of the resuspensions to obtain countable colonies. Transconjugant colonies were obtained either at undiluted or 10^1^ diluted resuspensions. Control, as well as every combination of donor + recipient + single other species were plated in two types of selective plates: LB agar (Sigma) with 30 µg/ml kanamycin and DAP and CHROMagar (Condalab) with 100 µg/ml ampicillin; which were used to obtain CFU/ml counts of ꞵ3914 donor strain, and to distinguish *E. coli* transconjugants from any other species able to grow in ampicillin; and on the nonselective plates made of CHROMagar (without DAP) (Condalab) to count *E. coli* recipient isolates. They were left overnight at 37°C and colonies were counted the next day.

The pOXA-48 plasmid conjugation rate was estimated using the formula: $T/( {D \cdot R \cdot \Delta t} )$ (Lopatkin et al. 2016, Huisman et al. 2022) where the CFU/ml of the transconjugants is represented by *T; D* are CFU/ml the donor, and *R* are CFU/ml of the recipient. The time in which conjugation took place is represented by *Δt*, and it was always 1 h; the approximate time needed for pOXA-48 to produce transconjugants (León-Sampedro et al. 2021), while keeping on-plate growth to a minimum.

### Conjugation rates of pOXA-48 to uropathogenic *E. coli* in artificial urine spent media

**Figure 1.**
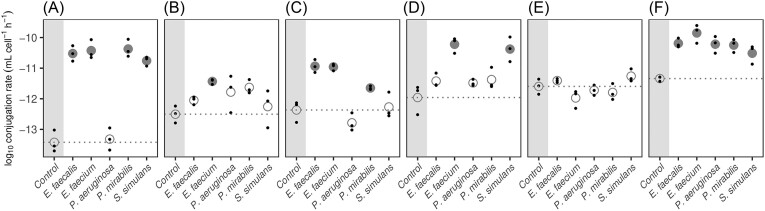
Conjugation rates of pOXA-48 plasmid to six uropathogenic *E. coli* recipient strains (A–F) in the absence (control) and presence of one additional UTI community member (*E. faecium, E. faecalis, S. simulans, P. aeruginosa*, and *P. mirabilis*). Small dots indicate individual replicate measurements (*n* = 3), large dots replicate means. Community members with a significant impact on conjugation rates relative to the basal conjugation rate (control, dotted line) are indicated by solid circles (ANOVA, Dunnett’s test, *P* < .05).

To assess the indirect interaction effect of the enterococci isolates, via metabolic compounds in their exudates (de Vos et al. 2017), on the conjugation efficiency of *E. coli*, conjugation experiments between donor strain ꞵ3914 and two *E. coli* isolates; B and F (Fig. 1), were performed on conditioned media agar plates containing spent medium from two enterococcal isolates, and the controls on AUM agar plates. The two *E. coli* isolates were chosen based on contrasting conjugation rates (Fig. 1). The protocol followed the same methodology as in the previous section; namely, mixing together 50 µl of each culture at an OD600 ratio of 5:1, plating the full droplet in the middle of the plates and incubating at 37°C for 1 h; with the main difference being the plate composition.

Specifically, spent media were recovered from two UTI isolates; *E. faecalis* and *E. faecium* by inoculating bacterial glycerol stocks in 200 ml 1x AUM in Erlenmeyer flasks shaking at 200 rpm at 37°C for 48 h. Afterwards, cultures were distributed into 50 ml culture tubes and centrifuged for 15 min at 4800 × *g* at room temperature. The resulting supernatants were filtered twice with bottle filter tops; 0.45 μm and 0.2 μm filters, respectively. To make sure that all bacteria were filtered out, spent medium was plated on CHROMagar plates, and incubated at 37°C for 24 h, whereafter the plates showed no bacterial growth.

The conditioned media agar plates were prepared using 50% Micro agar (15 g/l) (Duchefa Biochemie), 25% spent media, and 25% 3x AUM (including 1x concentration AUM salts). Control plates did not contain spent media in and consisted of 50% Micro agar (15 g/l), 25% 1x AUM, and 25% 3x AUM (including 1x concentration AUM salts). The end concentration of AUM in the conditioned media plates depended on how much nutrients were depleted by the bacteria, between 0,75x AUM (if all nutrients were consumed) and 1x AUM depending (if no nutrients were consumed). All plates contained 300 μM DAP to ensure the survival of donor bacteria.

### Statistical analysis

Statistical analyses were performed in R v4.1.2 (R Core Team 2021) and the package DescTools v0.99.50 (Signorell 2024). We used log_10_-transformed conjugation rates for analyses after visually confirming normality and homoscedasticity of the transformed data (Supplementary Figs 2 and 3). We tested the differences in basal conjugation rates of pOXA-48 plasmid to different *E. coli* recipient strains with a one-way ANOVA (*P* < .05). To assess the impact of community members (Conjugation rates of pOXA-48 to uropathogenic *E. coli* with and without community members on AUM) and exudates of community members (Conjugation rates of pOXA-48 to uropathogenic *E. coli* in artificial urine spent media) on conjugation rates, we first tested whether the effect of *E. coli* recipient strain and community member were independent (two-way ANOVA, *P* < .05). Because we found a significant interaction between the two main effects, we proceeded to assess the impact of community members on conjugation rates associated with each recipient strain separately (one-way ANOVA followed by Dunnett’s test for comparison to control, *P* < .05).

## Results

To investigate the impact of other community members often coisolated from polymicrobial UTIs on the conjugation efficiency of uropathogenic *E. coli*, we compared the pOXA-48 reception rate through conjugation of isolated uropathogenic *E. coli* with that of uropathogenic *E. coli* in the presence of other species commonly isolated from polymicrobial UTIs.

### Efficiency of uropathogenic *E. coli* to take up pOXA-48 plasmid via conjugation in LB

Specifically, we performed conjugation experiments between *E. coli* donor strain ꞵ3914 and nine uropathogenic *E. coli* isolates, using plasmid pOXA-48; initially on LB media (see the section ‘Materials and methods’). Three out of these nine *E. coli* isolates didn't take up the plasmid. Of the six uropathogenic *E. coli* isolates that did take up the plasmid (Fig. 1 A–F, see the section ‘Materials and methods’), they did so with different conjugation efficiencies; indicated by the qualitative assessment of the maximum dilution that obtained transconjugants (Supplementary Table 1).

### Conjugation rates of pOXA-48 to uropathogenic *E. coli* with and without community members on AUM

Of the six uropathogenic *E. coli* isolates that could take up the plasmid, we tested the differential effects on the conjugation rates with donor strain ꞵ3914 in the absence and in the presence of each of five other species frequently coisolated in polymicrobial UTIs in AUM: *E. faecium, E. faecalis, S. simulans, P. aeruginosa*, and *P. mirabilis*. Conjugation rate experiments were performed on AUM agar by bringing donor, recipient, and one other isolate of the above-mentioned species, together in a droplet of conjugation mix, as described by León-Sampedro et al. (2021), DelaFuente et al. (2022), and Alonso-del Valle et al. (2023) (see the section ‘Materials and methods’).

We first analyzed the two main effects (*E. coli* isolates and individual community members) across the whole experiment. We found that the effect of community members differs significantly for different *E. coli* isolates (two-way ANOVA, Df = 25, *F* value = 14.15, interaction: *P* < 2e-16). Moreover, we found that the basal conjugation rates, in the absence of community members, differ significantly between the six *E. coli* isolates (ANOVA, Df = 5, *F* value = 15.82, *P* = 6.4e-05). The conjugation rates of pOXA-48 from donor to recipient in isolation range within two orders of magnitude between the different isolates; from 4.7 × 10^−14^ for *E. coli* isolate A, to 4.6 × 10^−12^ for *E. coli* isolate F (Fig. 1). Assessing the effect of the bacterial interactions on the conjugation rate, we did not find that any of the other tested isolates inhibit the growth of either donor or recipient to such an extent that the variation in donors and recipients alone cannot explain the increase in conjugation rate (Supplementary Table 2). Only for *E. coli* isolates B and F, the changes of donors and recipients in the presence of *E. faecium* can partially explain the statistically significant changes in conjugation rate (Fig. 1, one-way ANOVA and Dunnett’s *post hoc* test, *P* < .05).

Rather, we found that bacterial interactions generally have a positive effect on the conjugation rates. Particularly the Gram-positive species *E. faecium* and *E. faecalis*, but also *S. simulans* contribute to this effect. For five of the six *E. coli* isolates, at least one *Enterococcus* species has a significant positive effect on the conjugation rate, whereas *S. simulans* has a positive effect on the conjugation rates in three of the *E. coli* isolates. The Gram-negative species *P. aeruginosa* and *P. mirabilis* generally have a less prominent effect. *P. mirabilis* alters the conjugation rates in three of the six *E. coli* species, whereas *P. aeruginosa* only affects the conjugation rates in one *E. coli* isolate (Fig. 1; Supplementary Table 2).

The extent to which pairwise interactions changed the conjugation rates varied substantially between isolates. For instance, for *E. coli* isolate A, three UTI community members increased the conjugation rate by three orders of magnitude. For isolate C, the presence of both *Enterococcus* species increased the conjugation rate by two orders of magnitude. Yet, for *E. coli* isolate E we could not detect any significant changes to the conjugation rate due to the influence of any other of the UTI community members (Fig. 1). The magnitude of the variability of the conjugation rates was constant throughout the tested conditions (Supplementary Fig. 1).

We find that the conjugation rate levels for the tested isolates do not correspond to their assigned phylogroups (Croxall et al. 2011a) (Supplementary Table 3). For instance, isolates A and D are both part of ECOR group B2 (Ochman and Selander 1984) as well as two of the isolates that did not take up the plasmid (based on the efficiencies of uropathogenic *E. coli* to take up pOXA-48 plasmid via conjugation in LB). One of the latter two belongs to the sequence type 131, a drug-resistant uropathogenic strain of *E. coli* widely disseminated among both community and hospital patients (Lau et al. 2008). This suggests that specific genetic components other than phylogroup classification are important for the ability to take up the pOXA-48 plasmid.

### Conjugation rates of pOXA-48 to uropathogenic *E. coli* in artificial urine spent media

To investigate if the increased conjugation rates, particularly due to the presence of enterococci, were due to their exudates (for instance metabolic products produced), we tested whether the presence of spent media of *E. faecium* and *E. faecalis* can recapitulate the findings. Conjugation experiments were performed with two *E. coli* isolates (isolates B and F) on spent medium agar plates (see the section ‘Materials and methods’), with the spent media from the two enterococci. These isolates were chosen based on the contrasting conjugation rates from Fig. 1.

**Figure 2.**
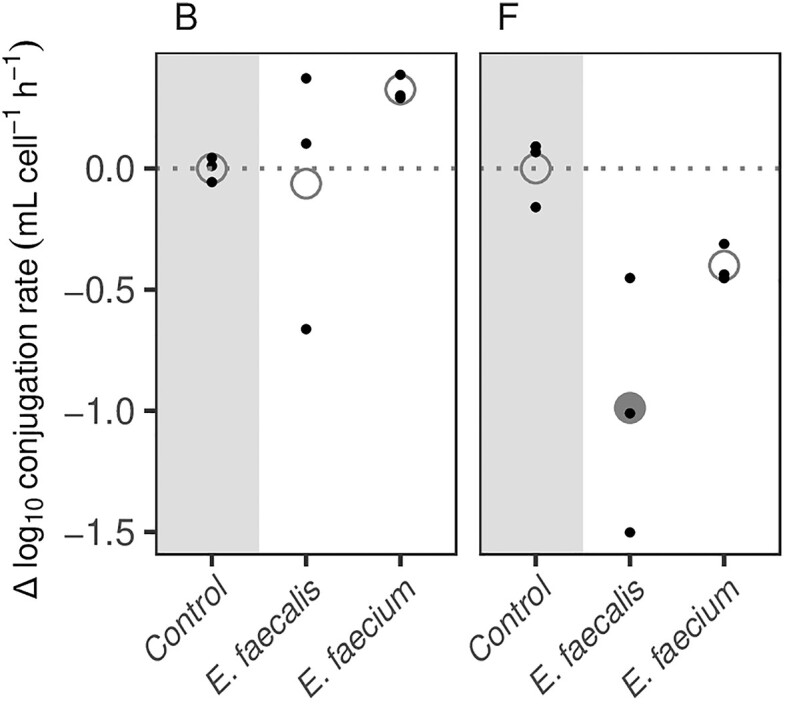
Relative differences in conjugation rates of pOXA-48 plasmid into two *E. coli* recipient strains (B and F) in the absence (Control) and presence of spent media containing exudates from another UTI community member (*E. faecium* and *E. faecalis)*. Small dots indicate individual replicate measurements, large dots replicate means. One replicate measurement (*E. coli* F *with E. faecalis*) was below the detection limit and was set to 1e-14 for the analysis. Community members with a significant impact on conjugation rates (solid circles) relative to the control conjugation rate (dotted line) are indicated by solid circles (ANOVA, Dunnett’s test, *P* < .05).

We found that the spent media experiment generally shows a rather small negative effect on the conjugation rate. Namely, in three combinations of *E. coli* under the influence of enterococci: *E. coli* B with *E. faecalis* and *E. faecium*, and *E. coli* F with *E. faecium* the effect was not significant and in one; *E. coli* F in the spent medium of *E. faecalis*, the effect was significant but negative. Therefore, this experiment cannot explain the marked positive effect on the conjugation rate in the presence of *E. faecium* and *E. faecalis* (Fig. 2; Supplementary Table 4) compared to the positive effects found in the AUM experiments. We have also tested these results under additional reference conditions aimed at assessing the boundary conditions of nutrient depletion in the conditioned medium. Supplementary Fig. 4 shows that under these conditions, the relative effect of enterococci on the conjugation rates of pOXA-48 plasmid into two *E. coli* isolates B and F, was significant and negative in three combinations: *E. coli* B with *E. faecalis* and *E. coli* F with *E. faecalis* and *E. faecium*, while for *E. coli* B with *E. faecium* the effect was not significant. This indicates that primary or secondary metabolites released by *E. faecium* and *E. faecalis* are unlikely to be the leading cause of the increased conjugation rates observed in the coculture experiments.

It should be noted that Gram-positive enterococci hardly grow in AUM (10^3^–10^4^ CFU/ml), these relatively low counts mimic their growth in urine (Flores-Mireles et al. 2015). These cells are therefore present in low numbers in the coculture conjugation rate experiments on the AUM agar plates. Spent medium agar plates containing exudates from these cells therefore likely contain a higher concentration of these metabolic exudates compared to the concentration of these exudates in the drops in the coculture conjugation experiments. We cannot rule out that these different concentrations in the different experiments may influence the effect on conjugation rates. That we do observe a marked increase in conjugation rates in the physical presence of enterococci, but not in the presence of their exudates, suggests that Gram-positive bacteria affect the conjugative transfer of AMR in uropathogenic *E. coli*, in a manner that is likely dependent on the physical interaction of *E. coli* and the Gram-positive species.

## Discussion

This work is aimed at investigating the impact of species often coisolated from polymicrobial UTIs on the conjugation efficiency of uropathogenic *E. coli*. We show that these species can positively affect the transfer of pOXA-48 plasmid from *E. coli* to uropathogenic *E. coli* in the low-nutrient AUM after 1 h of conjugation. Gram-positive species, particularly enterococci, and also sometimes Gram-negative species, such as *P. aeruginosa* and *P. mirabilis* increase the conjugation rates of pOXA-48 between *E. coli*.

Nine uropathogenic *E. coli* isolates were initially selected for conjugation with pOXA-48. Only six of these *E. coli* isolates took up the plasmid, as verified by PCR with pOXA-48 specific primers (see the section ‘Materials and methods’). The fact that each of them was associated with unique basal conjugation rates indicates that there are host-dependent genetic background interactions that determine these rates, which is in accordance with other findings (Alonso-del Valle et al. 2023, Benz and Hall 2023). Regarding the different sequence types and phylogroups assigned to the tested *E. coli* isolates, we find that the levels of conjugation rates, and even the ability to take up pOXA-48 plasmid, were not consistent with those. These results contrast previous literature showing that specific phylogroups are associated with specific transferability of plasmids (Carattoli 2011), that are associated with particular pathogenic phenotypes or multidrug resistance, as is the case with sequence type 131 (Lau et al. 2008).

In general, the presence of a plasmid in a new bacterial host might come with fitness costs, for which single compensatory mutations are often sufficient to completely ameliorate such costs, suggesting that these are caused by specific genetic conflicts rather than generic properties of plasmids, such as their size, metabolic burden, or gene expression level (Hall et al. 2021). For instance, plasmid-encoded extended-spectrum beta-lactamase gene acquisition in diverse *E. coli* lineages can drive strain-specific interactions, such as chromosomal mutations affecting metabolic and regulatory functions that ultimately might affect plasmid stability and conjugation efficiency (Carrilero et al. 2023). In the wider context of bacterial communities, both the cost of plasmid carriage and its long-term maintenance in a focal strain are found to depend on the presence of competitors, and these interactions are species-specific too (Sünderhauf et al. 2023).

The conjugation rate experiments were performed in the low-nutrient AUM to recapitulate an environment that is closer to the *in vivo* environment of the uropathogens. Otherwise, similar conjugation experiments are often performed in LB (Alonso-del Valle et al. 2021, León-Sampedro et al. 2021, DelaFuente et al. 2022) or viande-levure (Card et al. 2017, Duxbury et al. 2021), which are rather rich media and yield mostly higher conjugation rates. The conjugation rates we find are similar to conjugation rates of pOXA-48 in one strain of *E. coli*, cultured under anaerobic conditions and in rather poor M9 minimal media (León-Sampedro et al. 2021). We, therefore hypothesize that the similarly low basal conjugation rates result from the low nutrient environment. This hypothesis was confirmed by testing the ability to take up pOXA-48 plasmid through conjugation via the rather crude qualitative droplet-droplet method in a rich LB medium (see the section ‘Materials and methods’; Supplementary Table 1).

Conditioned medium experiments indicate that metabolic compounds in the exudates of the cocultured enterococcal species are unlikely to be the leading cause of the increased transfer. Thus, *E. coli* growth and survival mediated by such exudates of enterococci are also unlikely to be involved (Keogh et al. 2016). Moreover, to limit such potential growth effects we performed the incubation step of the conjugation rate experiments for only 1 h, whereas other studies often use longer incubation times (Alonso-del Valle et al. 2023). And lastly, because the conjugation rate is calculated based on the number of donors, recipients and transconjugants at the end of the 1-h conjugation incubation time (after the cogrowth of the donor ꞵ3914, recipient *E. coli* and community member on the agar plate), we conclude that growth rate differences are not the cause of the marked increase in conjugation rate in the presence of these species.

Proximity between cells, such as that found in biofilms, is known to facilitate the spread of antibiotic resistance by promoting HGT (Fux et al. 2005) e.g. via quorum sensing, which leads to changes in gene expression, potentially expediting the acquisition of antibiotic resistance (Schroeder et al. 2017, Lin et al. 2021). However, if the increased conjugation rates would be caused by quorum sensing molecules released by enterococci, then we expected to observe an increase in conjugation rates in the presence of conditioned medium prepared from enterococci, and that was not the case. Our findings therefore suggest that the nature of the coculture and its direct interactions are important for these increased conjugation rates in AUM. Specifically, we hypothesize that physical contact or proximity (Stalder and Top 2016), between these species may play a role. It is worth mentioning that pOXA-48 is an Inc L/M plasmid type that encodes short, rigid pili known to have higher transfer frequencies on solid surfaces compared to liquid (Bradley 1980). Various types of cell-to-cell contact have been shown to be involved in promoting the transfer of genetic material within species (Morawska and Kuipers 2022). One alternative hypothesis would be that the presence of some Gram-positive species is strengthening the interaction between the two *E. coli* strains (donor and recipient) as a sort of defense mechanism, limiting the direct interaction of the Gram-positive ‘intruder’ with the interacting *E. coli* strains. This is reminiscent of biofilm formation as a defense mechanism (Donlan and Costerton 2002, Kumar et al. 2017).

The Gram-negative species *P. mirabilis*, also had a positive effect on the conjugation efficiency for three of the *E. coli* isolates. It is known that *P. mirabilis* raises the pH of the AUM medium (Broomfield et al. 2009, Chen et al. 2012, de Vos et al. 2017), and pH has been shown to influence conjugation rates (Alderliesten et al. 2020). Additionally, it may be that this raised pH leads to a stress response; stress responses are speculated to affect the conjugation rates (Johnsen and Kroer 2007). Additionally, we speculate that a potential explanation for *E. coli* strain-specific responses in conjugation efficiency in the presence of UTI community members is related to the fact that some of the *E. coli* strains have different colony morphologies (more or less fuzzy or rounded), which potentially relates with these isolates being more or less ‘sticky’ than others. These may, therefore, be more prone to proximate interactions, which are related with increased uptake of plasmids (Robledo et al. 2022). For future studies, it would be of great interest to understand how multispecies interactions would affect conjugation efficiencies in *E. coli*.

Although successful plasmid spread depends upon a balance between plasmid fitness effects on the host and rates of horizontal transmission (Duxbury et al. 2021), the fact that uropathogenic *E. coli* are conjugatable at these levels in an AUM environment suggests that the urinary tract and its urobiome, is a potential location where HGT takes place (Wolfe and Brubaker 2019, Jones et al. 2021, Kuznetsova et al. 2022, Montelongo Hernandez et al. 2022).

Finally, our findings on the increased conjugation rates of pOXA-48 to uropathogenic *E. coli* in the presence of, particularly, Gram-positive species underscore that ecological interactions are relevant for the conjugative transfer of AMR, also in a urine-like environment.

## Supplementary Material

xtae023_Supplemental_Files
