# Supplementary material for: Community context influences the conjugation efficiency of Escherichia coli
Source: FEMS Microbes. 2024 Jul 27;5:xtae023. doi: 10.1093/femsmc/xtae023 (PMC11338288; doi:10.1093/femsmc/xtae023)
Supplement: xtae023_Supplemental_Files [file xtae023_supplemental_files.zip › One_sentence_summary.docx]

Our study unveils the community impact on the conjugation efficiency of uropathogenic *E. coli* isolates, with Gram-positive species, particularly enterococci, enhancing the conjugation efficiency.
