# Supplementary material for: Community context influences the conjugation efficiency of Escherichia coli
Source: FEMS Microbes. 2024 Jul 27;5:xtae023. doi: 10.1093/femsmc/xtae023 (PMC11338288; doi:10.1093/femsmc/xtae023)
Supplement: xtae023_Supplemental_Files [file xtae023_supplemental_files.zip › FEMSMC-2024-003.R3 supplementary data - reviews and author response.docx]

**Reviews of manuscript FEMSMC-2024-003.r3**

**Reviewer 1: Sarah Duxbury, University of Warwick**

**Recommendation: Minor Revision**

This study seeks to understand the role of microbial community members of polymicrobial urinary tract infections (UTIs) on conjugation efficiencies of *Escherichia coli* strain pairs, investigating transfer of the beta-lactamase encoding resistance plasmid pOXA-48 to a set of clinical isolates of *E. coli*. The specific question addressed was to investigate the role of bacterial interactions on conjugation, specifically the role of Gram-positive bacterial species that are understudied in the context of UTIs and antimicrobial resistance. To address this question, the authors performed co-culture conjugation experiments between a labelled donor *E. coli* strain and a set of six clinical *E. coli* isolates that were able to take up the plasmid. These experiments were performed in the presence and absence of individual addition of five members of polymicrobial UTIs, consisting of both Gram-positive and Gram-negative bacterial species. Following an initial qualitative test of donor-recipient conjugation efficiencies in the absence of community members on LB agar, quantitative conjugation assays were performed on artificial urine medium (AUM) agar plates to mimic the urinary tract nutrient conditions. Assays were performed over a short time period (1 hour) to measure conjugation efficiency in the absence of growth-mediated effects. To test the role of microbial exudates on the conjugation efficiencies, a selection of conjugation assays were performed on agar plates supplemented with spent media from UTI isolates that influenced conjugation efficiencies.

The main finding of this study is that Gram-positive Enterococci isolated from polymicrobial UTIs can generally increase *E. coli – E. coli* conjugation efficiency of pOXA-48. This study also shows that basal conjugation efficiencies are influenced by *E. coli* strain, suggesting a role for genotypic background. Conjugation mediated effects appear to be independent of microbial exudates, suggesting a role for physical cell contacts influencing conjugation efficiencies, particularly as conjugation assays were performed in a spatially-structured environment. These findings are particularly interesting as fold increases in conjugation rates in the presence of community members can be as great as two orders of magnitude, differing widely depending on the identity of the recipient strain. Outstanding questions remain to discern genetic determinants of community-mediated effects relating to *E. coli* recipient strain identity, natural variation in conjugation-mediated effects across a larger set of clinical isolates and in the presence of other species and their combinations, in the context of polymicrobial UTIs. Additionally, the role of physical inter-species contacts on conjugation efficiencies remains to be determined.

Major concerns

A major concern that I have is on statistical support for the main conclusions of the paper and this requires further review in the Results and Methods.

1. Figure 1 highlights significant results as solid circles but test results are missing from the text. An ANOVA test was performed to compare log-transformed conjugation rates between all treatment conditions (presumably per *E. coli* pair), however the question being addressed was whether conjugation efficiencies differed between the control treatment and each of the treatments of individual community members, rather than investigating all pairwise comparisons between treatments. I would therefore recommend Dunnett’s tests for post-hoc comparisons of each of the treatment groups with the control group.

2. A separate ANOVA test should also be performed to statistically compare basal conjugation rates (control condition) between each *E. coli* recipient strain, supporting biological effects described in the text.

3. To back up the conclusion on lines 265-266, I would suggest an additional statistical analysis where the effects of *E. coli* isolate and treatment (presence and absence of different community members) and their interaction on conjugation efficiency can be tested, using a two-way ANOVA.

4. Analysis and data presentation in Supplementary Figure 3 requires review, including removing the correlation line, as sample size is small therefore statistical power is low for this analysis.

5. Figure 2 needs re-working. I would recommend to present data in the same way as in Figure 1, plotting all data points and overlying mean points to show the true data spread. Statistical analysis is needed to support the results of no effect in either direction, for conjugation efficiencies measured in the presence of exudates, normalised by control conditions. It appears that the exudates could be causing a decrease rather than an increase in conjugation rates, which is not discussed. Presentation of raw data in a Supplementary table would be helpful. The authors should also justify whether conclusions from this experiment can be compared with results from Figure 1, given the differences in experimental set-up. Exudates were dispersed within the agar medium rather than inoculated in the drop containing the *E. coli* pair, therefore concentrations may differ.

6. Lines 217 – 221: please justify use of statistical tests. This should include details of the diagnostic checks made for the assumptions and validity of data transformation.

Further methods or results details are required to justify conclusions.

1. This section on ‘Conjugation on LB’ on lines 105 -132 needs further detail and clarification as currently there is insufficient detail for this assay to be replicated and to understand how results were generated, including appropriate use of controls. Similarly, use of controls should be described in the section on conjugation with community members on AUM. Reciprocal plating of donor and recipient strain monocultures on each of the selective agars described on lines 190 – 192 as well as lack of growth/distinction from community members is needed. Explain use of CHROMagar plates and comparison with LB plates used for the donor strain. Could these different agar types have contributed to growth differences between donor and recipient backgrounds? Were *E. coli* isolates selectively counted, rather than community members that may also have taken up the plasmid? Lines 111 – 112: re-wording needed. Lines 113-117: Assay set-up needs clarification. Were stationary rather than exponential phase cultures used? Please justify use of a 5:1 donor:recipient ratio. Clarify how mixing was performed and set up of controls, including replication. Were droplets added as single drops or spread over the agar surface? Justify 4-hour duration for conjugation. Lines 118-119: re-wording and further details needed for biomass removal and washing steps. Lines 120 -123: please clarify that a set of serial 10-fold dilutions were performed. Lines 124-128: clarify type of agar plate- was this a square dish? Can a reference be added for this ‘running drop’ assay? Were selective and non-selective LB agar plates used? Please clarify how transconjugants were specifically selected.

2. Lines 182-183: please explain use of replication. Were these biological replicates performed as experiments on separate days, using different overnight cultures of donor, recipient and community members? Are these three replicates likely to be representative?

3. Lines 311-315: conclusions not supported through data presentation. Please provide data in the Results section for growth of Enterococci.

Minor concerns:

1. Abstract. Some textual editing is needed for further clarification, particularly to describe that the polymicrobial aspect is being tested by measuring conjugation rates in the presence of individual community members. Lines 9 and 10: clarify and motivate this sentence further. Specify that different bacterial species are being referred. Line 16: remove ‘interestingly’. Abbreviations on lines 13 and 20 need clarifying. Describe AUM abbreviation. Line 19: please specify more clearly the ‘nature and co-culture of the interactions’.

2. Introduction. Bridging between background and the knowledge gap should be clarified in lines 38 – 48 and the specific aims and objectives stated more clearly on lines 49 - 50. The influences of abiotic and biotic factors on HGT should be more clearly specified, separating out what is known and unknown. On lines 39 – 40, specify what is meant by ‘environment’, clarify how conjugation can be ‘limited by bacterial diversity’ on line 44 and what is meant by ‘microbial context’.

3. Figure 1 colour gradient is not needed as the conjugation rate values are presented in the y-axis. The legend should explain the use of lines on the plot, as this is unclear. Are these line connectors between the conjugation rate under control conditions and each mean value under community treatment conditions? These could be misinterpreted as error bars, therefore obscuring the biological effects so I would suggest to remove these. Sample sizes and replication should be stated in the figure legend.

4. Text description within the Results section should be written clearer to communicate key messages, including on lines 224-225, 236, 240 (community member included within the same drop), 245 (‘any of the other tested isolates’), 247 (remove ‘interestingly’), 259-260 (‘other community members’), 261.

5. General comment: keep consistent with measure of conjugation, as ‘efficiency’ rather than ‘rate’.

6. Lines 82- 83: specify that these isolates were used as recipients in the conjugation assays.

7. Lines 134-135: correct to plasmid transfer ‘to’ the six *E. coli* isolates (also on line 154), as these were recipients. Describe DNA extraction kit or method used.

8. Lines 155 and 164-165: clarify to describe that *E. coli- E. coli* conjugation is being measured, rather than plasmid transfer to community members. Line 158: make it clear that community members were being added individually to the *E. coli – E. coli* conjugation assay, rather than as a five species community.

9. Lines 166-167: describe how OD600 measurements were taken, using a spectrophotometer or plate reader. Line 168 and 170-171: please justify use of this ratio. Were OD values compared to cell densities? Line 175: please describe total volumes of donor-recipient-community member cultures.

10. Line 217: cite the version of R that was used, rather than RStudio.

11. The first paragraph of the discussion requires a re-write to summarise the main contributions of this study. In particular, the key finding that Gram-positive Enterococci can increase *E. coli- E. coli* conjugation efficiency needs to be discussed in more depth, highlighting the originality and significance of this finding. Positive effects of Gram-negative species on the conjugation efficiency for some of the *E. coli* pairs should also be highlighted and discussed, as well as potential explanation for *E. coli* strain-specific responses in conjugation efficiency in the presence of community members. Combined effects of the five members in a polymicrobial UTI environment should also be discussed.

12. Lines 303-304: VL medium is used to mimic the chicken gut environment. Please cite the following reference instead of Huisman et al., 2022. Card RM, Cawthraw SA, Nunez-Garcia J, Ellis RJ, Kay G, Pallen MJ, Woodward MJ, Anjum MF. An In Vitro Chicken Gut Model Demonstrates Transfer of a Multidrug Resistance Plasmid from Salmonella to Commensal Escherichia coli. mBio. 2017 Jul 18;8(4):e00777-17. doi: 10.1128/mBio.00777-17.

13. Line 311: remove ‘interestingly’. Line 318: population size effects not tested in this study.

14. Line 326: please define what is meant by the ‘nature and co-culture of the interactions’. Lines 326 – 334: please also comment on the role of spatial structure, as conjugation assays were performed in drops on agar rather than in liquid culture. How could this have affected conjugation efficiency and inter-species interactions? What was the significance of performing assays on agar? This type of plasmid is classified as an Inc L/M type, encoding short, rigid pili such that transfer is greater on surfaces than in liquid (Bradley, 1980).

15. Supplementary Figure 2 is not needed as data is repeated from Figure 1.

16. Check references for consistent style, including capitalisation of journal article titles and italicising species names.

**Reviewer 2**

**Recommendation: Minor Revision**

The manuscript by Bustamante and colleagues examines the influence of concomitant bacterial taxa on the transfer of plasmids between Enterobacterales, using Escherichia coli as a model. This is a significant issue that has been overlooked until now. The experimental design is appropriate, and the findings are presented clearly, although some possible limitations are also highlighted. Some comments are given for the author´s consideration.

Major comments

• The sample used (donor, receptors and concomitant bacterial species) should be described in detail to fully understand the results obtained. First, pOXA48 is a highly conserved and promiscuous IncL plasmid widely disseminated. The authors could explain why they chose the E. coli-E. coli primary matings model for the experiments (pOXA-IncL is more common among Klebsiella and non-E. coli enterobacterales than among E. coli). Second, provide information about the strains used as receptors (phylogroup, ST, virulence profile, plasmid content). Finally, the same for all the “concomitant” species uses (ie. Enterococcus faecium, Enterococcus faecalis). This point is relevant to interpreting the results since the authors insist on the relevance of the genotype for the heterogeneous results obtained.

• Did the authors sequence the plasmids in receptors and the transconjugants? This information could help to understand the differences in the basal conjugation (successful acquisition) rates.

• Line 287-288. The authors hypothesized that the interaction between enterococci and E. coli could be behind the increased conjugation rates. This suggestion should be discussed and better argued.

• Lines 331-334. Pheromone-responsive conjugation has been demonstrated only for Enterococcus faecalis. Change enterococcus for “ E. faecalis” in the text. Why do the authors extend the possibility of aggregation to other species? From the text, the reader can interpret that cell-to-cell enterococcal mechanisms could affect other species present. This is extremely speculative, and the sentence should be softened.

• The authors focus all the argumentation on transfer (conjugation rates). However, discussing differences in the successful plasmid acquisition/ stability in different receptors would be welcome.

• Enterococcus is a frequent cause of infection/co-infection because antibiotic treatments favour the selection of these organisms with are intrinsically tolerant/resistant to many first-line antibiotics (i.e. beta-lactams, aminoglycosides, macrolides,…). A tempting possibility is that this bacterial co-infection is a byproduct of antibiotic treatment, and sub-inhibitory antibiotic concentrations might trigger conjugation. This article would prove that other factors independent of antibiotic treatment influence the conjugation.

Other comments.

• Complete the species name in the title.

• Line 20. Describe the meaning of the acronym AUM

• Line 200-215. The sections “spent media” and “conjugation plates for spent media analysis” can be combined in a unique section about “spent media analysis”. This approach should be supported by reference. A brief explanation of the purpose of the analysis will be very welcome for non-expert readers.

• Figure 2. Specify to which E. coli isolates correspond to each result (see previous comment about the relevance of specifying each E. coli genotype).

• Revise italics for the bla genes.

**Editor Decision: Minor Revision**

**Author's Response to Decision Letter for (FEMSMC-2024-003)**

We would like to thank the reviewers for taking the time to provide constructive feedback, comments, and recommendations to our manuscript. We have addressed and incorporated the suggestions and recommendations by the reviewers, which have contributed to the improvement of the manuscript.

Below we have listed our response to the reviewers.

**Reviewer: 1**

Comments to the Author

This study seeks to understand the role of microbial community members of polymicrobial urinary tract infections (UTIs) on conjugation efficiencies of *Escherichia coli* strain pairs, investigating transfer of the beta-lactamase encoding resistance plasmid pOXA-48 to a set of clinical isolates of *E. coli*. The specific question addressed was to investigate the role of bacterial interactions on conjugation, specifically the role of Gram-positive bacterial species that are understudied in the context of UTIs and antimicrobial resistance. To address this question, the authors performed co-culture conjugation experiments between a labeled donor *E. coli* strain and a set of six clinical *E. coli* isolates that were able to take up the plasmid. These experiments were performed in the presence and absence of individual addition of five members of polymicrobial UTIs, consisting of both Gram-positive and Gram-negative bacterial species. Following an initial qualitative test of donor-recipient conjugation efficiencies in the absence of community members on LB agar, quantitative conjugation assays were performed on artificial urine medium (AUM) agar plates to mimic the urinary tract nutrient conditions. Assays were performed over a short time period (1 hour) to measure conjugation efficiency in the absence of growth-mediated effects. To test the role of microbial exudates on the conjugation efficiencies, a selection of conjugation assays were performed on agar plates supplemented with spent media from UTI isolates that influenced conjugation efficiencies.

The main finding of this study is that Gram-positive Enterococci isolated from polymicrobial UTIs can generally increase *E. coli – E. coli* conjugation efficiency of pOXA-48. This study also shows that basal conjugation efficiencies are influenced by *E. coli* strain, suggesting a role for genotypic background. Conjugation mediated effects appear to be independent of microbial exudates, suggesting a role for physical cell contacts influencing conjugation efficiencies, particularly as conjugation assays were performed in a spatially-structured environment. These findings are particularly interesting as fold increases in conjugation rates in the presence of community members can be as great as two orders of magnitude, differing widely depending on the identity of the recipient strain. Outstanding questions remain to discern genetic determinants of community-mediated effects relating to *E. coli* recipient strain identity, natural variation in conjugation-mediated effects across a larger set of clinical isolates and in the presence of other species and their combinations, in the context of polymicrobial UTIs. Additionally, the role of physical inter-species contacts on conjugation efficiencies remains to be determined.

Major concerns

A major concern that I have is on statistical support for the main conclusions of the paper and this requires further review in the Results and Methods.

We thank the reviewer for constructive suggestions on increasing the robustness of our statistical analysis. As described in more detail below, we followed all suggestions for additional/modified tests, rewrote the Methods accordingly, and added the results to the main text. All new tests corroborated our initial findings and interpretations.

1. Figure 1 highlights significant results as solid circles but test results are missing from the text. An ANOVA test was performed to compare log-transformed conjugation rates between all treatment conditions (presumably per *E. coli* pair), however the question being addressed was whether conjugation efficiencies differed between the control treatment and each of the treatments of individual community members, rather than investigating all pairwise comparisons between treatments. I would therefore recommend Dunnett’s tests for post-hoc comparisons of each of the treatment groups with the control group.

We followed the reviewer's suggestions, modified the figure, and used Dunnett’s test for post hoc significance testing against the controls instead of testing all pairs with Tukey-Kramer. Through the increased power of Dunnett’s test, one additional combination of *E. coli* recipient and community member (*E. coli* with *P. mirabilis*) now shows a significant increase in conjugation rates. We have updated the Methods and Results section accordingly.

We have updated the results section accordingly in lines 291-307: ‘We first analyzed the two main effects (*E. coli* isolates and individual community members) across the whole experiment. We found that the effect of community members differs significantly for different *E. coli* isolates (two-way ANOVA, interaction: p < 2e-16). Moreover, we found that the basal conjugation rates, in the absence of community members, differ significantly between the six *E. coli* isolates (ANOVA, p = 6.4e-05). The conjugation rates of donor and recipient in isolation range within two orders of magnitude between the different isolates; from 4.7 x 10^-14^ for *E. coli* isolate A, to 4.6 x 10^-12^ for *E. coli* isolate F (Figure 1). Assessing the effect of the bacterial interactions on the conjugation rate, we did not find that any of the other tested isolates inhibit the growth of either donor or recipient (Supplementary Table 2).

Rather, we found that bacterial interactions generally have a positive effect on the conjugation rates. Particularly the Gram-positive species *E. faecium* and *E. faecalis*, but also *S. simulans* contribute to this effect. For five of the six *E. coli* isolates, at least one *Enterococcus* species has a significant positive effect on the conjugation rate, whereas *S. simulans* has a positive effect on the conjugation rates in three of the *E. coli* isolates. The Gram-negative species *P. aeruginosa* and *P. mirabilis* generally have a less prominent effect. *P. mirabilis* alters the conjugation rates in two of the six *E. coli* species, whereas *P. aeruginosa* only affects the conjugation rates in one *E. coli* isolate (Figure 1; Supplementary Table 2)’.

2. A separate ANOVA test should also be performed to statistically compare basal conjugation rates (control condition) between each *E. coli* recipient strain, supporting biological effects described in the text.

We carried out the suggested test and found that basal conjugation rates are significantly different among recipient *E. coli* strains (ANOVA, p-value 6.4e-05). We’ve added the respective information to both Methods and Results sections.

3. To back up the conclusion on lines 265-266, I would suggest an additional statistical analysis where the effects of *E. coli* isolate and treatment (presence and absence of different community members) and their interaction on conjugation efficiency can be tested, using a two-way ANOVA.

We carried out a two-way ANOVA, as suggested, and found a significant interaction between recipient strains and individual community members, supporting our conclusion that the effect of the community member depends on the specific recipient strains. We have updated the text in lines 264-272: ‘We tested the differences in basal conjugation rates of different *E. coli* recipient strains with a one-way ANOVA (p <0.05). To assess the impact of community members (Conjugation rates of pOXA-48 to uropathogenic *E. coli* with and without community members on AUM experiment) and exudates of community members (Conjugation rates of pOXA-48 to uropathogenic *E. coli* in spent media experiment) on conjugation rates, we first tested whether the effect of *E. coli* recipient strain and community member were independent (two-way ANOVA, p < 0.05). Because we found a significant interaction between the two main effects, we proceeded to assess the impact of community members on conjugation rates for each recipient strain separately (one-way ANOVA followed by Dunnett’s test for comparison to control, p < 0.05)’.

4. Analysis and data presentation in Supplementary Figure 3 requires review, including removing the correlation line, as sample size is small therefore statistical power is low for this analysis.

We agree with the reviewer, the distribution of basal conjugation rates does not have a large sample size, nor is the sampling uniformly distributed (only one isolate with a very low conjugation rate, others exhibit larger conjugation rates). Therefore, we do agree with the reviewer that we cannot make a correct statistical inference about the relationship between basal conjugation rates and effect of the other bacterial species. We would need more data to test this specific hypothesis correctly, therefore we have decided to remove this figure S3 from the manuscript.

5. Figure 2 needs re-working. I would recommend to present data in the same way as in Figure 1, plotting all data points and overlying mean points to show the true data spread. Statistical analysis is needed to support the results of no effect in either direction, for conjugation efficiencies measured in the presence of exudates, normalized by control conditions. It appears that the exudates could be causing a decrease rather than an increase in conjugation rates, which is not discussed.

We followed the reviewer's suggestion and present the data now analogous to Experiment/Figure 1 and we use the same statistical test approach: two-way ANOVA with indicated significant interaction, which we follow up with recipient-specific one-way ANOVAs and Dunnett’s post hoc test.

We have also updated the text referring to this figure, ‘We found that the spent media experiments generally show a rather small negative effect on the conjugation rate. Namely, in three experiments the effect was not significant and in one; *E. coli* in the spent medium of *E. faecalis,* the effect was significant but negative. Therefore, these experiments cannot explain the marked positive effect on the conjugation rate in the presence of *E. faecium* and *E. faecalis* (Figure 2; Supplementary Table 3) compared to the positive effects found in the AUM experiments’, lines 328-333.

Presentation of raw data in a Supplementary table would be helpful.

We thank the reviewer for the suggestion. We have created Supplementary Table 3 with the raw data from the ‘Conjugation rates of pOXA-48 to uropathogenic *E. coli* in spent media’ experiments. We have referred to it in the text: ‘We found that the spent media experiments generally show a rather small negative effect on the conjugation rate. Namely, in three experiments the effect was not significant and in one; *E. coli* in the spent medium of *E. faecalis,* the effect was significant but negative. Therefore, these experiments cannot explain the marked positive effect on the conjugation rate in the presence of *E. faecium* and *E. faecalis* (Figure 2; Supplementary Table 3) compared to the positive effects found in the AUM experiments’ in lines 328-333.

The authors should also justify whether conclusions from this experiment can be compared with results from Figure 1, given the differences in experimental set-up. Exudates were dispersed within the agar medium rather than inoculated in the drop containing the *E. coli* pair, therefore concentrations may differ.

We thank the reviewer for this remark. We agree that indeed, the concentrations of exudates in the spent media plates from the enterococcal species may be higher than in the droplets of the co-culture experiment. Arguably, it would be logical to hypothesize that the effect on the conjugation rates would be higher in the spent media experiments, yet this happens only in co-culture.

Linking the reviewer's remarks on statistics (above) and this particular remark, we have thoroughly looked at this experimental dataset again, and have decided to omit data from strain A, as this had a larger spread in the basal conjugation rates compared to all other experiments performed. The main findings from these particular experiments are however still intact, we do not find the marked increase in conjugation rate in the presence of spent media of *E. faecium* and *E. faecalis*. Despite that, the concentration of exudates in these conditioned media plates is higher than in the droplets.

We have also eluded better to this in the result section of the text in lines 335-346: ‘It should be noted that Gram-positive enterococci hardly grow in artificial urine medium (10^3^-10^4^ CFU/ml), these relatively low counts mimic their growth in urine [(Flores-Mireles *et al.* 2015)](https://www.zotero.org/google-docs/?E0nztD). These cells are therefore present in low numbers in the co-culture conjugation rate experiments on the AUM agar plates. Spent medium agar plates containing exudates from these cells therefore likely contain a higher concentration of these metabolic exudates compared to the concentration of these exudates in the drops in the co-culture conjugation experiments. We cannot rule out that these different concentrations in the different experiments may influence the effect on conjugation rates. That we do observe a marked increase in conjugation rates in the physical presence of enterococci, but not in the presence of their exudates, suggests that Gram-positive bacteria affect the conjugative transfer of AMR in uropathogenic *E. coli*, in a manner that is likely dependent on the physical interaction of *E. coli* and the Gram-positive species’.

6. Lines 217 – 221: please justify use of statistical tests. This should include details of the diagnostic checks made for the assumptions and validity of data transformation.

We updated our statistical analyses according to the previous reviewer's suggestions and now discuss them in more detail in the Materials and methods section. We have added supplementary plots (Supplementary Figure 2 and Supplementary Figure 3) supporting our data assumptions and the validity of transformation (normality, homoscedasticity). Particularly, we have updated our Statistical analysis subsection, now from lines 261-272, as follows: ‘Statistical analyses were performed in R v4.1.2 and the package DescTools v0.99.50. We used log_10_-transformed conjugation rates for analyses after visually confirming normality and homoscedasticity of the transformed data (Supplementary Figure 2 and Supplementary Figure 3). We tested the differences in basal conjugation rates of different *E. coli* recipient strains with a one-way ANOVA (p <0.05). To assess the impact of community members (Conjugation rates of pOXA-48 to uropathogenic *E. coli* with and without community members on AUM experiment ) and exudates of community members (Conjugation rates of pOXA-48 to uropathogenic *E. coli* in spent media experiment) on conjugation rates, we first tested whether the effect of *E. coli* recipient strain and community member were independent (two-way ANOVA, p < 0.05). Because we found a significant interaction between the two main effects, we proceeded to assess the impact of community members on conjugation rates for each recipient strain separately (one-way ANOVA followed by Dunnett’s test for comparison to control, p < 0.05)’.

Further methods or results details are required to justify conclusions.

1. This section on ‘Conjugation on LB’ on lines 105 -132 needs further detail and clarification as currently there is insufficient detail for this assay to be replicated and to understand how results were generated, including appropriate use of controls.

We thank the reviewer for the constructive comments on this section. We have clarified this section by incorporating the suggestions and the addition of more details for the correct replication of the assay, including the use of controls and selective media used. The updated section is now titled: ‘Conjugation rates of pOXA-48 to uropathogenic *E. coli* on LB’; described in lines 120-156.

Similarly, use of controls should be described in the section on conjugation with community members on AUM.

Thank you for your suggestion. We have reworded the text from lines 205-207: ‘For the assessment of the basal conjugation rates of *E. coli*, 50 µL of donor and 50 µL of recipient were added and gently mixed in a 0.5 mL tube, preserving a 5:1 OD600 ratio. This combination was the control of the experiment; the basal conjugation rate’. This means that our controls for the conjugation rate experiments of pOXA-48 to uropathogenic *E. coli* with and without community members on AUM were the combination of donor and recipient, i.e. the basal conjugation rate.

Reciprocal plating of donor and recipient strain monocultures on each of the selective agars described in lines 190 – 192 as well as lack of growth/distinction from community members is needed.

Thank you for this remark. We have updated the text to make it clear that donor and *E. coli* **transconjugants** were recovered from the selective plates. *E. coli* recipients were recovered from non-selective CHROMagar plates. Community members were not counted, as they were not the focal species of our study and they were not expected to grow in plates containing ampicillin, given that they were sensitive to this antibiotic; and less resistant in the case of PAO1. The updated text is in lines 224-231: ‘Transconjugant colonies were obtained either at undiluted or 10^1^ diluted re-suspensions. Control, as well as every combination of donor + recipient + single other species were plated in two types of selective plates: LB agar (Sigma) with 30 µg/mL kanamycin and DAP and CHROMagar (Condalab) with 100 µg/mL ampicillin; which were used to obtain CFU/mL counts of ꞵ3914 donor strain, and to distinguish *E. coli* transconjugants from any other species able to grow in ampicillin; and on the non-selective plates made of CHROMagar (without DAP) (Condalab) to count *E. coli* recipient isolates. They were left overnight at 37°C and colonies were counted the next day’.

Explain use of CHROMagar plates and comparison with LB plates used for the donor strain. Could these different agar types have contributed to growth differences between donor and recipient backgrounds? Were *E. coli* isolates selectively counted, rather than community members that may also have taken up the plasmid?

Regarding the use of CHROMagar and LB plates: throughout our experiments, we have not seen any indication that this contributed to differences in the formation of colonies on these plates. Additionally, our results don’t reflect any growth differences between the donor and the recipient strains in 1 hour of conjugation. This can be corroborated in the CFU/mL counts from Supplementary Table 2, showcasing that donor and recipients were recovered in the same orders of magnitude throughout the experiments.

Referring to the second question, *E. coli* transconjugants were indeed selectively counted in the CHROMagar plates containing ampicillin, where we expected transconjugants. Therefore, the community members were not counted. Enterococci for instance, grew in the rather narrow ‘halo’ around the *E. coli* transconjugant colonies, whereas *P. aeruginosa* PAO1 had a wider range. We are following-up on these particular community-conjugation-antibiotic growth interactions in our subsequent research.

Lines 111 – 112: re-wording needed.

We have reworded the sentence to: ‘The next day, 3 independent colonies were picked from each isolate and grown overnight in 2 mL of LB at 37°C and continuous shaking at 200 rpm. Donor cultures were grown with 30 µg/mL kanamycin and 300 µM DAP’ in lines 126-128.

Lines 113-117: Assay set-up needs clarification. Were stationary rather than exponential phase cultures used?

We thank the reviewer for pointing out this need for clarification. We have updated the text to: ‘The following day, the overnight cultures, which were at that time in stationary phase, were mixed…’ in line 129.

Please justify the use of a 5:1 donor:recipient ratio.

We appreciate this remark. We have updated the text to justify the use of this ratio: ‘The following day, the overnight cultures, which were at that time in stationary phase, were mixed in a 5:1 donor-to-recipient volume ratio. This relationship was established after testing several donor-to-recipient proportions, and this ratio resulted as the most effective one for plasmid transfer’ in lines 129-132.

Clarify how mixing was performed and set up of controls, including replication. Were droplets added as single drops or spread over the agar surface?

We thank the reviewer for this suggestion. We have updated the text to include the use of replication and how the droplets were handled: ‘The experiment was performed in triplicates; 50 µL of the donor culture and 10 µL of the recipient culture were gently mixed by pipetting in 0.5 mL tubes. The full 60 µL droplets were spotted in the middle of LB agar (Sigma) plates without antibiotics but with 300 µM DAP (Sigma)’ in lines 132-134.

Regarding controls, the text now states: ‘The controls consisted of 60 µL of isolated cultures of donor and recipient; each in triplicates, which were also spotted in the middle of individual LB agar (Sigma) plates with 300 µM DAP (Sigma)’ in lines 139-141.

Justify 4-hour duration for conjugation.

We thank the reviewer for pointing out the need to justify the use of 4 hours for conjugation. We have updated the text to: ‘The full 60 µL droplets were spotted in the middle of LB agar (Sigma) plates without antibiotics but with 300 µM DAP (Sigma), and left to air-dry in the flow cabin, after which they were incubated at 37°C for 4 hours to recover transconjugants. This mating time is short enough to reduce the chances of secondary conjugation events from transconjugants to recipients and the impact of potential differences in donor, recipient, and transconjugant growth rates on conjugation frequency determination [(Alonso-del Valle *et al.* 2023)](https://www.zotero.org/google-docs/?zYdEYv)’ in lines 133-139.

Lines 118-119: re-wording and further details needed for biomass removal and washing steps.

Thanks for this suggestion, as it is important for readers to understand that we gently recovered the transconjugants. We have edited the text to: ‘After incubation, a metal loop was used to scoop out the biomass of each of the droplets, which were immediately washed and resuspended by pipetting in tubes containing 2 mL of sterile 0.9% NaCl solution’ in lines 142-144.

Lines 120 -123: please clarify that a set of serial 10-fold dilutions were performed.

We agree with this need for further clarification of the protocol. We have added this to the text: ‘These were further diluted in serial ten-fold dilutions from 10^1^ until 10^7^ using a 96-well plate’ in line 144-145.

Lines 124-128: clarify type of agar plate- was this a square dish?

We agree with the need for clarification. We have added to the text: ‘A multichannel pipette was used to transfer 10 µL from each dilution at the first quarter of a round agar plate’ in lines 147-148.

Can a reference be added for this ‘running drop’ assay?

We thank the reviewer for the question. We have at the beginning of the section, cited the source from which we adapted the protocol. It is in lines 121-123: ‘This protocol was used to assess, rather qualitatively, the ability of the uropathogenic *E. coli* isolates to take up pOXA-48 plasmid, and it was adapted from [(Alonso-del Valle *et al.* 2021, 2023)](https://www.zotero.org/google-docs/?mlQjqp)’.

Were selective and non-selective LB agar plates used? Please clarify how transconjugants were specifically selected.

We agree with the need to clarify how the transconjugants were specifically selected. The text was updated to: ‘Every donor and recipient mix, as well as the controls, were plated on transconjugant-selective LB agar plates with ampicillin 100 µg/mL and on LB agar plates with kanamycin 30 µg/mL (Sigma) without DAP, to make sure that neither donor nor recipients in isolation would grow’ in lines 149-152.

2. Lines 182-183: please explain use of replication. Were these biological replicates performed as experiments on separate days, using different overnight cultures of donor, recipient and community members? Are these three replicates likely to be representative?

We thank the reviewer for pointing out the need for further explanation of the use of replication. Therefore, in lines 185-189 we have written: ‘Each conjugation experiment was performed in a single day and consisted of six assays: the ‘basal conjugation rate’ assay with only donor and recipient; and the pairwise ‘community member assays’ where each of the five other species were added individually to the donor and recipient combination. Every assay was performed with three biological replicates’.

We would like to clarify that the protocol for conjugation rate experiments presented in this study was optimized over several months using triplicates in every experiment to be able to perform conjugation experiments in a low nutrient environment, in only one hour. After optimization we were succeeded in obtaining transconjugants after only one hour of incubation, and we consistently observed that, for instance, the basal conjugation efficiency of strain A always had a very low conjugation efficiency, compared to the other strains (sometimes even below our detection limit in this one-hour time span). Or for example, that strain F always had a very high conjugation efficiency compared to the other isolates. Another constant observation was the striking effect of enterococci on the conjugation rates compared to the basal (in isolation) conjugation rates of the *E. coli* isolates. Thus, given the consistent results obtained with the use of triplicates in every experiment, during optimization of the protocol and in its application, we do believe that they are representative of the results presented in this study.

3. Lines 311-315: conclusions not supported through data presentation. Please provide data in the Results section for growth of Enterococci.

We thank the reviewer for this suggestion, and we agree that the statement should be moved to a more prominent place as the Results section; and we have done so. In lines 335-346, the text has been updated to: ‘It should be noted that Gram-positive enterococci hardly grow in artificial urine medium (103-104 CFU/ml), these relatively low counts mimic their growth in urine [(Flores-Mireles *et al.* 2015)](https://www.zotero.org/google-docs/?E0nztD). These cells are therefore present in low numbers in the co-culture conjugation rate experiments on the AUM agar plates. Spent medium agar plates containing exudates from these cells therefore likely contain a higher concentration of these metabolic exudates compared to the concentration of these exudates in the drops in the co-culture conjugation experiments. We cannot rule out that these different concentrations in the different experiments may influence the effect on conjugation rates. That we do observe a marked increase in conjugation rates in the physical presence of enterococci, but not in the presence of their exudates, suggests that Gram-positive bacteria affect the conjugative transfer of AMR in uropathogenic *E. coli*, in a manner that is likely dependent on the physical interaction of *E. coli* and the Gram-positive species’.

Minor concerns:

1. Abstract. Some textual editing is needed for further clarification, particularly to describe that the polymicrobial aspect is being tested by measuring conjugation rates in the presence of individual community members.

We appreciate the suggestion made by the reviewer. We have edited the text in the abstract and it is now explicit that the community members were added individually. This is particularly the case in lines 14-17: ‘Pairwise conjugation rate experiments were conducted between a donor *E. coli* strain containing the pOXA-48 plasmid and six uropathogenic *E. coli* isolates, in the presence and absence of five different species commonly co-isolated in polymicrobial UTIs to elucidate their effect on the conjugation efficiency of *E. coli*’.

Lines 9 and 10: clarify and motivate this sentence further. Specify that different bacterial species are being referred to.

We have edited the text in the abstract to make this specific point: ‘In urinary tract infections, different bacteria can live in a polymicrobial community consisting of different species’. It is now in lines 10-11.

Line 16: remove ‘interestingly’.

We agree with this suggestion from the reviewer, we have removed this word from the appointed line.

Abbreviations on lines 13 and 20 need clarifying.

We have updated the abbreviation on line 13 as: ‘pOXA-48 plasmid’ now in line 15. And from previous line 20, now in line 23, to: ‘artificial urine medium’.

Describe AUM abbreviation.

We thank the reviewer for the remark. We have changed the text to: ‘artificial urine medium’ and it is now in line 23.

Line 19: please specify more clearly the ‘nature and co-culture of the interactions’.

We thank the reviewer for pointing this out. There has been a syntax mistake and we have corrected it accordingly. What we meant to say is that: ‘We hypothesize that the nature of the co-culture and physical interactions are important for these increased conjugation rates in an artificial urine medium environment’. This is now in lines 21-23.

2. Introduction. Bridging between background and the knowledge gap should be clarified in lines 38 – 48

Thank you for your suggestion on this need for clarification. We have updated the text between lines 42-55: ‘Although the ecology and functioning of microbial communities are typically studied in one specific environment at a time [(Smillie *et al.* 2011)](https://www.zotero.org/google-docs/?I4rb8i), it is known that the rate of HGT is strongly dependent on the abiotic and the biotic environments [(Sessitsch *et al.* 2023)](https://www.zotero.org/google-docs/?6ayZFg). For instance, resource availability and temperature [(Pallares-Vega *et al.* 2021)](https://www.zotero.org/google-docs/?KYuSTx) or other abiotic factors such as salt stress [(Beuls *et al.* 2012)](https://www.zotero.org/google-docs/?8DVC2g), can significantly affect the rate at which HGT via conjugation occurs. Biotic factors, such as the presence of ecological interaction partners, can affect the spread of conjugative plasmids within and between host species [(Bottery 2022)](https://www.zotero.org/google-docs/?cADYah). Additionally, the horizontal transmission of plasmids can be limited by bacterial diversity due to the ‘dilution effect’; i.e. the phenomenon whereby living alongside less proficient host species reduces the expected infection risk for a focal host species [(Kottara *et al.* 2021)](https://www.zotero.org/google-docs/?SdoVLE). The microbial context, such as the presence of competitors, can also determine the cost and benefits of conjugative plasmid maintenance [(Sünderhauf *et al.* 2023)](https://www.zotero.org/google-docs/?gdCIJd). Moreover, ecological interactions can alter factors such as growth rate and population densities, which together can affect the cost of plasmid carriage and conjugation rates [(Duxbury *et al.* 2021)](https://www.zotero.org/google-docs/?hk7LT5)’.

and the specific aims and objectives stated more clearly on lines 49 - 50.

We thank the reviewer for pointing out the need for clarification of the specific aims and objectives of our study. We have edited the text from lines 56-60 to: ‘It is still an open question to what extent bacterial interactions affect the transfer of antibiotic resistance by HGT via conjugation in bacterial communities. Given that complex communities are difficult to study, we investigate this question for a simple and tractable, yet relevant, system consisting of bacterial species often co-isolated from elderly patients diagnosed with urinary tract infections (UTIs) [(Croxall *et al.* 2011)](https://www.zotero.org/google-docs/?JtLVJI)’.

The influences of abiotic and biotic factors on HGT should be more clearly specified, separating out what is known and unknown. On lines 39 – 40, specify what is meant by ‘environment’, clarify how conjugation can be ‘limited by bacterial diversity’ on line 44 and what is meant by ‘microbial context’.

We appreciate these suggestions by the reviewer. We have specified what is meant by ‘environment’, ‘limited by bacterial diversity’ and ‘microbial context’ from lines 42 to 53 in the text: ‘Although the ecology and functioning of microbial communities are typically studied in one specific environment at a time [(Smillie *et al.* 2011)](https://www.zotero.org/google-docs/?I4rb8i), it is known that the rate of HGT is strongly dependent on the abiotic and the biotic environments [(Sessitsch *et al.* 2023)](https://www.zotero.org/google-docs/?6ayZFg). For instance, resource availability and temperature [(Pallares-Vega *et al.* 2021)](https://www.zotero.org/google-docs/?KYuSTx) or other abiotic factors such as salt stress [(Beuls *et al.* 2012)](https://www.zotero.org/google-docs/?8DVC2g), can significantly affect the rate at which HGT via conjugation occurs. Biotic factors, such as the presence of ecological interaction partners, can affect the spread of conjugative plasmids within and between host species [(Bottery 2022)](https://www.zotero.org/google-docs/?cADYah). Additionally, the horizontal transmission of plasmids can be limited by bacterial diversity due to the ‘dilution effect’; i.e. the phenomenon whereby living alongside less proficient host species reduces the expected infection risk for a focal host species [(Kottara *et al.* 2021)](https://www.zotero.org/google-docs/?SdoVLE). The microbial context, such as the presence of competitors, can also determine the cost and benefits of conjugative plasmid maintenance [(Sünderhauf *et al.* 2023)](https://www.zotero.org/google-docs/?gdCIJd)’.

3. Figure 1 **colour** gradient is not needed as the conjugation rate values are presented in the y-axis. The legend should explain the use of **lines** on the plot, as this is unclear. Are these line connectors between the conjugation rate under control conditions and each mean value under community treatment conditions? These could be misinterpreted as error bars, therefore obscuring the biological effects so I would suggest to remove these. **Sample sizes and replication** should be stated in the figure legend.

We appreciate the suggestion from the reviewer. We have updated Figure 1 by removing the color gradient and the vertical lines. The caption of this figure now states: ‘Figure 1. Conjugation rates of six *E. coli* recipient strains (A-F) in the absence (control) and presence of one additional UTI community member (*E. faecium*, *E. faecalis*, *S. simulans, P. aeruginosa* and *P. mirabilis)*. Small dots indicate individual replicate measurements (n=3), large dots replicate means. Community members with a significant impact on conjugation rates relative to the basal conjugation rate of each *E. coli* recipient strain (control, dotted line) are indicated by solid circles (ANOVA, Dunnett’s test, p < 0.05)’ in lines 317-322.

4. Text description within the Results section should be written clearer to communicate key messages, including on lines 224-225, 236, 240 (community member included within the same drop), 245 (‘any of the other tested isolates’),

We appreciate this suggestion from the reviewer. We have updated the text in the results section to make these point clear, particularly from lines 284-290: ‘...we tested the differential effects on the conjugation efficiency of six uropathogenic *E. coli* isolates with donor strain ꞵ3914 in the absence and in the presence of each of five other species frequently co-isolated in polymicrobial UTIs in AUM: *E. faecium*, *E. faecalis*, *S. simulans, P. aeruginosa* and *P. mirabilis*. Conjugation rate experiments were performed on AUM agar by bringing donor, recipient and one other isolate of the above-mentioned species, together in a droplet of conjugation mix, as described by [(León-Sampedro *et al.* 2021; DelaFuente *et al.* 2022; Alonso-del Valle *et al.* 2023)](https://www.zotero.org/google-docs/?jDfR6i) (Materials and Methods)’.

247 (remove ‘interestingly’),

We agree with this suggestion from the reviewer, we have removed this word from the appointed line.

259-260 (‘other community members’), 261.

We have updated the text, now in lines 311-313, to make clear what the effects of each UTI community isolate was on *E. coli* isolate E: ‘Yet, for *E. coli* isolate E we could not detect any significant changes to the conjugation rate due to the influence of any other of the UTI community members (Figure 1)’.

5. General comment: keep consistent with measure of conjugation, as ‘efficiency’ rather than ‘rate’.

Thanks for this suggestion, to clarify any confusion that may arise from the use of ‘efficiency’ versus ‘rates’ we have added the sentence: ‘It is important to mention that the term conjugation ‘efficiency’ is used as a qualitative description of the ability of recipient *E. coli* isolates to uptake pOXA-48 plasmid. On the other hand, conjugation ‘rate’ is used to refer to the quantitative assessment of the transfer of pOXA-48 plasmid to uropathogenic *E. coli*’, at lines 85-88.

We prefer to use the rather qualitative description of efficiencies for uptake of the plasmid in the uropathogenic *E. coli*, while the rate refers to the quantitative assessment (as measured in the experiments) of the transfer of the plasmid from the donor to the recipient.

6. Lines 82- 83: specify that these isolates were used as recipients in the conjugation assays.

We thank the reviewer for the suggestion. We have corrected the text, and it is now specified that: ‘Initial conjugation experiments aimed at testing their ability to take up pOXA-48 plasmid resulted in six final uropathogenic *E. coli* isolates that were used as recipients in pairwise mating assays in the presence of UTI community members’ in lines 93 and 96.

7. Lines 134-135: correct to plasmid transfer ‘to’ the six *E. coli* isolates (also on line 154), as these were recipients.

We appreciate this remark from the reviewer. We have updated the text accordingly. In line 158 it is now stated: ‘To confirm plasmid transfer to the six *E. coli* isolates, and in line 179: ‘...plasmid transfer to the six *E. coli* isolates’.

Describe DNA extraction kit or method used.

We thank the reviewer for this remark. We would like to mention that the DNA extraction method used in the DNA extraction and PCR section, from lines 157-179, is specifically referred to in lines 158-159: ‘DNA was extracted using a previously in-house developed ultra-fast DNA extraction method for *E.coli* described in Brons et al. [(2020)](https://www.zotero.org/google-docs/?HWRc60)’.

8. Lines 155 and 164-165: clarify to describe that *E. coli- E. coli* conjugation is being measured, rather than plasmid transfer to community members.

We thank the reviewer for the suggestion, and we agree with the need for clarification. We have updated the text to specify that *E. coli* - *E. coli* conjugation was being measured in our experiments. This is now stated in lines 180-181: ‘Conjugation rates of pOXA-48 to uropathogenic *E. coli* with and without community members on AUM’ and also in lines 182-188: ‘We performed a quantifiable method based on [(León-Sampedro *et al.* 2021; DelaFuente *et al.* 2022; Alonso-del Valle *et al.* 2023)](https://www.zotero.org/google-docs/?sYckqV) to determine the conjugation of pOXA-48 from donor strain ꞵ3914 to six uropathogenic *E. coli* isolates in the presence and absence of five members of the polymicrobial UTI community in AUM media. Each conjugation experiment was performed in a single day and consisted of six assays: the ‘basal conjugation rate’ assay with only donor and recipient; and the pairwise ‘community member assays’ where each of the five other species were added individually to the donor and recipient combination’.

Line 158: make it clear that community members were being added individually to the *E. coli – E. coli* conjugation assay, rather than as a five species community.

We thank the reviewer for pointing out this need for clarification. We have incorporated in the text: ‘Each conjugation experiment was performed in a single day and consisted of six assays: the ‘basal conjugation rate’ assay with only donor and recipient; and the pairwise ‘community member assays’ where each of the five other species were added individually to the donor and recipient combination’ in lines 185-188.

9. Lines 166-167: describe how OD600 measurements were taken, using a spectrophotometer or plate reader.

We agree with this suggestion, and we have incorporated it to the text: ‘After 24 hours, optical density measurements at 600 nm (OD600) measurements were taken of all strains using a 1 ml culture in a disposable cuvette in a spectrophotometer’ in lines 195-196.

Line 168 and 170-171: please justify use of this ratio.

We thank the reviewer for pointing out the need for this justification. We have updated the text to: ‘Each culture was then further diluted to obtain a starting population size that, in combination, would maintain a 5:1:1 OD600 ratio between donor, recipient and community member, respectively. These proportions were the same as used for the ‘Conjugation rates of pOXA-48 to uropathogenic *E. coli* on LB’ protocol (Materials and methods). Specifically, the OD600 values used were 1, 0.2 and 0.2, respectively; except in the case of *E. faecium* and *E. faecalis*, where the OD was always lower than 0.2’ in lines 198-204.

Were OD values compared to cell densities?

We thank the reviewer for the question. We measured OD600 for each of the cultures for the set-up of the experiments, but we did not compare these values to cell densities in CFU/mL. What we did, however, was to calculate the conjugation rates based on the CFU/mL from the colony counts on each donor, recipient and transconjugant selective agar. This data is found on Table 2 from the supplementary data.

Line 175: please describe total volumes of donor-recipient-community member cultures.

We appreciate this remark by the reviewer, to make it more concrete, we have added this to the text: ‘Therefore, the total droplet volume for the basal conjugation assay was 100 µL and 150 µL for the individual community member assays’ in lines 213-214.

10. Line 217: cite the version of R that was used, rather than RStudio.

We agree with this suggestion from the reviewer. The updated text is: ‘Statistical analyses were performed in R v4.1.2’ in line 261.

11. The first paragraph of the discussion requires a re-write to summarize the main contributions of this study. In particular, the key finding that Gram-positive Enterococci can increase *E. coli- E. coli* conjugation efficiency needs to be discussed in more depth, highlighting the originality and significance of this finding.

Thank you for the suggestion. We have added a paragraph to the beginning of the discussion section that states: ‘This work is aimed at investigating the impact of species often co-isolated from polymicrobial UTIs on the conjugation efficiency of uropathogenic *E. coli.* We show that these species can positively affect the *E. coli* - *E. coli* conjugation efficiency in the low-nutrient artificial urine medium after 1 hour of conjugation. Gram-positive species, particularly enterococci, and also sometimes Gram-negative species, such as *P. aeruginosa* and *P. mirabilis* increase the conjugation rates between *E. coli*’ from lines 360 to 365.

Positive effects of Gram-negative species on the conjugation efficiency for some of the *E. coli* pairs should also be highlighted and discussed, as well as potential explanation for *E. coli* strain-specific responses in conjugation efficiency in the presence of community members. Combined effects of the five members in a polymicrobial UTI environment should also be discussed.

We thank the reviewer for these suggestions. We have updated the text from lines 410-421 to address each of them: ‘The Gram-negative species *P. mirabilis,* also had a positive effect on the conjugation efficiency for some of the *E. coli* isolates. It is known that *P. mirabilis* raises the pH of the AUM medium [(Broomfield *et al.* 2009; Chen *et al.* 2012; de Vos *et al.* 2017)](https://www.zotero.org/google-docs/?cd643f), and pH has been shown to influence conjugation rates [(Alderliesten *et al.* 2020)](https://www.zotero.org/google-docs/?KAGi53). Additionally, it may be that this raised pH leads to a stress response; stress responses are speculated to affect the conjugation rates [(Johnsen and Kroer 2007)](https://www.zotero.org/google-docs/?2Yngsz). Additionally, we speculate that a potential explanation for *E. coli* strain-specific responses in conjugation efficiency in the presence of UTI community members has to do with the fact that some of the *E. coli* strains have different colony morphologies (more or less fuzzy or rounded), which potentially relates with these isolates being more or less ‘sticky’ than others. These may therefore be more prone to proximate interactions which are related with increased uptake of plasmids [(Robledo *et al.* 2022)](https://www.zotero.org/google-docs/?AUh3XT). For future studies, it would be of great interest to understand how multispecies interactions would affect conjugation efficiencies in *E. coli*’.

12. Lines 303-304: VL medium is used to mimic the chicken gut environment. Please cite the following reference instead of Huisman et al., 2022. Card RM, Cawthraw SA, Nunez-Garcia J, Ellis RJ, Kay G, Pallen MJ, Woodward MJ, Anjum MF. An In Vitro Chicken Gut Model Demonstrates Transfer of a Multidrug Resistance Plasmid from Salmonella to Commensal *Escherichia coli*. mBio. 2017 Jul 18;8(4):e00777-17. doi: 10.1128/mBio.00777-17.

We thank the reviewer for this suggestion. We have changed the reference accordingly. It is now cited in line 384.

13. Line 311: remove ‘interestingly’.

We agree with this suggestion from the reviewer, we have removed this word from the appointed line.

Line 318: population size effects not tested in this study.

We have updated the text to make this point clear. With ‘population size effects’, we meant the effect of enterococci on the growth and survival of *E. coli.* In lines 383-386 it is now specified that: ‘Conditioned medium experiments indicate that metabolic compounds in the exudates of the co-cultured enterococcal species are unlikely to be the leading cause of the increased transfer. Thus, *E. coli* growth and survival mediated by such exudates of enterococci are also unlikely to be involved [(Keogh *et al.* 2016)](https://www.zotero.org/google-docs/?myrteh)’

14. Line 326: please define what is meant by the ‘nature and co-culture of the interactions’.

We thank the reviewer for pointing this out. There has been a syntax mistake and we have corrected it accordingly. What we meant to say is that: ‘Our findings suggest that the nature of the co-culture and its interactions are important for these increased conjugation rates in artificial urine medium’. This is now in lines 393-394.

Lines 326 – 334: please also comment on the role of spatial structure, as conjugation assays were performed in drops on agar rather than in liquid culture. How could this have affected conjugation efficiency and inter-species interactions? What was the significance of performing assays on agar? This type of plasmid is classified as an Inc L/M type, encoding short, rigid pili such that transfer is greater on surfaces than in liquid (Bradley, 1980).

We appreciate this suggestion about the role of spatial structure, and we have added it to the text, from lines 393-404: ‘Our findings suggest that the nature of the co-culture and its interactions are important for these increased conjugation rates in artificial urine medium. Specifically, we hypothesize that physical contact or proximity [(Stalder and Top 2016)](https://www.zotero.org/google-docs/?jK7LsI), potentially mediated by signaling molecules [(Lin *et al.* 2021)](https://www.zotero.org/google-docs/?tICJJA) between these species may play a role. It is worth mentioning that pOXA-48 is an Inc L/M plasmid type that encodes short, rigid pili known to have higher transfer frequencies on solid surfaces compared to liquid [(Bradley 1980)](https://www.zotero.org/google-docs/?wOpj8K). Various types of cell-to-cell contact have been shown to be involved in promoting the transfer of genetic material within species [(Morawska and Kuipers 2022)](https://www.zotero.org/google-docs/?gw3X1y). For example, the pheromone-inducible aggregation substance of *Enterococcus faecalis*, a virulence factor that promotes the aggregation and therefore proximity of *E. faecalis*, has been shown to have a positive effect on the conjugation rate of this species [(Waters and Dunny 2001)](https://www.zotero.org/google-docs/?hWFYZV). Potentially such enterococcal aggregation could also, as a by-product of its own aggregation, increase the proximity of other conjugating species, such as *E. coli*, in mixed cultures’.

15. Supplementary Figure 2 is not needed as data is repeated from Figure 1.

We agree with the reviewer. We have therefore removed the previous version of Supplementary Figure 2 from the supplementary data, and we have now replaced it for a relevant one. Specifically, the new caption states: ‘(A) Residuals histogram and (B) quantile-quantile plot of log10-transformed pOXA-48 conjugation rates with and without community members (related to Figure 1)’. This figure has been uploaded together with the other supplementary files for review.

16. Check references for consistent style, including capitalisation of journal article titles and italicising species names.

We thank the reviewer for pointing out these inconsistencies with the reference style. We have reviewed carefully that every reference cited is now uniformly written in the same format.

**Reviewer: 2**

Comments to the Author

The manuscript by Bustamante and colleagues examines the influence of concomitant bacterial taxa on the transfer of plasmids between Enterobacterales, using *Escherichia coli* as a model. This is a significant issue that has been overlooked until now. The experimental design is appropriate, and the findings are presented clearly, although some possible limitations are also highlighted. Some comments are given for the author´s consideration.

Major comments

• The sample used (donor, receptors and concomitant bacterial species) should be described in detail to fully understand the results obtained. First, pOXA48 is a highly conserved and promiscuous IncL plasmid widely disseminated. The authors could explain why they chose the *E. coli*-*E. coli* primary matings model for the experiments (pOXA-IncL is more common among Klebsiella and non-*E. coli* enterobacterales than among *E. coli*).

Thank you for pointing this out. It was precisely the fact that pOXA-48 is a promiscuous and widely disseminated plasmid, particularly in the clinical setting ([León-Sampedro *et al*. 2021](https://www.zotero.org/google-docs/?KVSikn)) that made it an interesting plasmid to test in our study. Our initial goal was to test if this plasmid would be able to be transferred at all to our *E. coli* uropathogenic isolates; once this was confirmed, we continued with our protocol optimization. The focal species in our study was always *E. coli.*

Second, provide information about the strains used as receptors (phylogroup, ST, virulence profile, plasmid content).

We appreciate the reviewer’s remark about characterizing the recipient strains used in this study. We agree that this information is relevant and will therefore be part of follow-up studies, potentially a prospective PhD student. For the scope of this study, however, we have not characterized our isolates yet. We can confirm however, that each of the *E. coli* strains used in this study were isolated from different elderly persons.

Finally, the same for all the “concomitant” species uses (ie. E. faecium, E. faecalis). This point is relevant to interpreting the results since the authors insist on the relevance of the genotype for the heterogeneous results obtained.

We thank the reviewer for pointing out the need to characterize the UTI community member species used in this study. Similar to what was appointed above regarding the *E. coli* isolates, we believe that this is important, and it will be part of future studies. We also used wild isolates in this case, except for PAO1, and we can confirm that they were different species coming from different patients.

• Did the authors sequence the plasmids in receptors and the transconjugants? This information could help to understand the differences in the basal conjugation (successful acquisition) rates.

We agree with the reviewer that sequencing the plasmids in recipients and transconjugants would help us understand the differences observed between the isolates in their basal conjugation rates. This is indeed a good suggestion for follow-up work. The scope of this study, however, was as a first exploration of the conjugative potential in a minimal community structure of the UTI *E. coli* isolates that we tested.

• Line 287-288. The authors hypothesized that the interaction between enterococci and *E. coli* could be behind the increased conjugation rates. This suggestion should be discussed and better argued.

We thank the reviewer for this suggestion. We have better explained our hypothesis in the text from lines 328-346: ‘We found that the spent media experiments generally show a rather small negative effect on the conjugation rate. Namely, in three experiments the effect was not significant and in one; *E. coli* in the spent medium of *E. faecalis,* the effect was significant but negative. Therefore, these experiments cannot explain the marked positive effect on the conjugation rate in the presence of *E. faecium* and *E. faecalis* (Figure 2; Supplementary Table 3) compared to the positive effects found in the AUM experiments. This indicates that primary or secondary metabolites released by *E. faecium* and *E. faecalis* are unlikely to be the leading cause of the increased conjugation rates observed in the co-culture experiments. It should be noted that Gram-positive enterococci hardly grow in artificial urine medium (10^3^-10^4^ CFU/ml), these relatively low counts mimic their growth in urine [(Flores-Mireles *et al.* 2015)](https://www.zotero.org/google-docs/?E0nztD). These cells are therefore present in low numbers in the co-culture conjugation rate experiments on the AUM agar plates. Spent medium agar plates containing exudates from these cells therefore likely contain a higher concentration of these metabolic exudates compared to the concentration of these exudates in the drops in the co-culture conjugation experiments. We cannot rule out that these different concentrations in the different experiments may influence the effect on conjugation rates. That we do observe a marked increase in conjugation rates in the physical presence of enterococci, but not in the presence of their exudates, suggests that Gram-positive bacteria affect the conjugative transfer of AMR in uropathogenic *E. coli*, in a manner that is likely dependent on the physical interaction of *E. coli* and the Gram-positive species’.

• Lines 331-334. Pheromone-responsive conjugation has been demonstrated only for Enterococcus faecalis. Change enterococcus for “ *E. faecalis*” in the text.

We agree with the reviewer. We have updated the text from lines 400 to 402: ‘For example, the pheromone-inducible aggregation substance of *Enterococcus faecalis*, a virulence factor that promotes the aggregation and therefore proximity of *E. faecalis*, has been shown to have a positive effect on the conjugation rate of this species [(Waters and Dunny 2001)](https://www.zotero.org/google-docs/?hWFYZV)’.

Why do the authors extend the possibility of aggregation to other species? From the text, the reader can interpret that cell-to-cell enterococcal mechanisms could affect other species present. This is extremely speculative, and the sentence should be softened.

We agree with the reviewer that the sentence should be softened. We have reworded it to: ‘For example, the pheromone-inducible aggregation substance of *Enterococcus faecalis*, a virulence factor that promotes the aggregation and therefore proximity of *E. faecalis*, has been shown to have a positive effect on the conjugation rate of this species [(Waters and Dunny 2001)](https://www.zotero.org/google-docs/?hWFYZV). Potentially such enterococcal aggregation could also, as a by-product of its own aggregation, increase the proximity of other conjugating species, such as *E. coli*, in mixed cultures’ in lines 400-404. Given the extent of the effect of enterococci on the conjugation rate from pOXA-48 to *E. coli,* we would like to keep this message.

• The authors focus all the argumentation on transfer (conjugation rates). However, discussing differences in the successful plasmid acquisition/ stability in different receptors would be welcome.

We thank the reviewer for this suggestion. We have referred to it in lines 422-426: ‘Although successful plasmid spread depends upon a balance between plasmid fitness effects on the host and rates of horizontal transmission [(Duxbury *et al.* 2021)](https://www.zotero.org/google-docs/?K6nGse), the fact that uropathogenic *E. coli* are conjugatable at these levels in an artificial urine medium environment suggests that the urinary tract and its urobiome, is a potential location where HGT takes place [(Wolfe and Brubaker 2019; Jones *et al.* 2021; Kuznetsova *et al.* 2022; Montelongo Hernandez, Putonti and Wolfe 2022)](https://www.zotero.org/google-docs/?NnPERh)’.

• Enterococcus is a frequent cause of infection/co-infection because antibiotic treatments favour the selection of these organisms which are intrinsically tolerant/resistant to many first-line antibiotics (i.e. beta-lactams, aminoglycosides, macrolides,…). A tempting possibility is that this bacterial co-infection is a byproduct of antibiotic treatment, and sub-inhibitory antibiotic concentrations might trigger conjugation. This article would prove that other factors independent of antibiotic treatment influence the conjugation.

We appreciate this comment, we fully agree.

Other comments.

• Complete the species name in the title.

We thank the reviewer for pointing this out. We have now updated the text to: ‘Community context influences the conjugation efficiency of *Escherichia coli*’ in line 1.

• Line 20. Describe the meaning of the acronym AUM

We thank the reviewer for pointing this out. We have now written the meaning of AUM in the text: ‘artificial urine medium’ in line 23.

• Line 200-215. The sections “spent media” and “conjugation plates for spent media analysis” can be combined in a unique section about “spent media analysis”.

We agree with the reviewer. We have merged the sections into one called: ‘Conjugation rates of pOXA-48 to uropathogenic *E. coli* in spent media’ and it is now found from lines 238-259.

This approach should be supported by reference. A brief explanation of the purpose of the analysis will be very welcome for non-expert readers.

We thank the reviewer for this suggestion. We have incorporated an explanation at the beginning of the section: ‘To assess the indirect interaction effect of the enterococci isolates, via metabolic compounds in their exudates [(de Vos *et al.* 2017)](https://www.zotero.org/google-docs/?1Xr6E1), on the conjugation efficiency of *E. coli*, conjugation experiments between donor strain ꞵ3914 and two *E. coli* isolates were performed on spent media agar plates from two enterococcal isolates, and the controls on AUM . The protocol followed the same methodology as in the previous section; namely, mixing together 50 µL of each culture at an OD600 ratio of 5:1, plating the full droplet in the middle of the plates and incubating at 37°C for 1 hour; with the main difference being the plate composition’, from lines 239 to 245.

• Figure 2. Specify to which E. coli isolates correspond to each result (see previous comment about the relevance of specifying each E. coli genotype).

We have updated Figure 2. Its caption, from lines 348-357, now states the following: ‘Figure 2. Relative differences in conjugation rates of two *E. coli* recipient strains (B, F) in the absence (Control) and presence of spent media containing exudates from another UTI community member (*E. faecium*, *E. faecalis)*. Small dots indicate individual replicate measurements, large dots replicate means. One replicate measurement (*E. coli* F *with E. faecalis*) was below the detection limit and was set to 1e-14 for the analysis. Community members with a significant impact on conjugation rates (solid circles) relative to the control conjugation rate (dotted line) are indicated by solid circles (ANOVA, Dunnett’s test, p < 0.05)’.

• Revise italics for the bla genes.

We appreciate this remark by the reviewer. We have removed italics for the bla genes, particularly the ‘oxa48’ part, throughout the manuscript.

Not related to reviewer comments: We would like to note that we have stated in the new version of the manuscript the use of *P. aeruginosa* strain PAO1 in the ‘Bacterial isolates’ subsection of the Materials and Methods, and specifically in the ‘Conjugation rates of pOXA-48 to uropathogenic *E. coli* with and without community members on AUM’ experiments. It is worth mentioning that this is not a UTI isolate, but a reference strain (received from Asheigh Griffin, University of Oxford). The reason for this was that the *P. aeruginosa* UTI isolates in our collection were resistant to ampicillin and PAO1 was less resistant and therefore better suitable for our experiments. We noticed that we had not declared this in the initial submission of our manuscript, and we wanted to clarify this point, not only in the text but also in this response to the reviewers.

**Reviews of manuscript FEMSMC-2024-003.r1**

**Reviewer 1: Sarah Duxbury, University of Warwick**

**Recommendation: Minor Revision**

I would like to thank the authors for their detailed and thorough revision of this manuscript addressing all reviewers’ comments. This has resulted in a greatly improved manuscript that highlights the key findings and novelty of this study.

There are some points still to raise however, requiring additional clarification.

Minor comments

1. Lines 328 – 329: please justify whether the potential for reduced nutrient concentration in the AUM spent media plates (0.75 x AUM) due to nutrient consumption, could have affected measured conjugation rates in Figure 2. Were additional control plates tested with 0.75 x AUM rather than 1 x AUM?

2. Lines 241 and 326: please justify in the text how the selection of *E. coli* isolates B and F was decided for the spent media assays. Were these isolates chosen based on contrasting basal conjugation rates shown in Figure 1? How does this link with the specific isolate results described on lines 309 – 313?

3. Lines 328 – 330: please specify these results shown in Figure 2 more clearly, differentiating between the results found for isolates B and F. It is not immediately clear that “in three experiments the effect was not significant” relates to the total of four experiments performed over the two isolates.

4. Lines 293 and 295: please report the full statistical results of the ANOVA tests including F statistic and degrees of freedom.

5. Les 298 – 299: please clarify how lack of inhibition of growth of the donor and recipient strains was inferred. Is this a qualitative assessment based on CFU/ml densities presented in Supplementary Table 2?

6. Lines 149 – 152: For the LB agar plates with kanamycin without DAP, please clarify why transconjugant selection was expected. Is kanamycin resistance also present and transferred on the pOXA-48 plasmid?

7. Line 261: include citations and references for R and the package DescTools.

8. Line 400: abbreviate the genus name for *Enterococcus faecalis*.

9. Line 247: capitalise the ‘E’ for Erlenmeyer.

**Reviewer 2**

**Recommendation: Major Revision**

This is my second review of the paper written by Bustamante et al. The authors analyzed the effect of concomitant bacterial taxa on the “conjugation rate”/”conjugation efficiency” of plasmids between Escherichia coli strains. The goal is to explore how often plasmid transferability is triggered by other species that coexist in polymicrobial infections, taking as a model strains from polymicrobial urinary tract infections (ITU) in the elderly. Different factors associated with plasmid acquisition, transfer, or maintenance have been extensively studied. However, the impact of concomitant species is still a neglected issue.

The authors have notably improved the original version of the manuscript by adding statistics and clarifying some technical details of the protocols. However, some reviewer´s comments on interpreting the results have not been fully addressed. Some additional comments are given for the author´s consideration.

Firstly, this referee considers it relevant to describe the lineage of the E. coli strains used. The authors believe that they may be out of the scope of this paper and might be included in future studies. I disagree with this opinion. Plasmid cost/transferability is often strain/lineage-specific; thus, the genotypic background can influence the "conjugation efficiency." Different Escherichia coli phylogroups contain different plasmid backgrounds, which could influence the uptake and transferability of incoming plasmids. Also, in the absence of any genotyping, one can not be sure how similar these E. coli strains are (pandemic clones are widespread in both hospital and community-based settings, and they often vary in the accessory genome). I understand the authors do not wish to include more experiments, but the description of the sample seems to be relevant for interpreting the results of this work. This point should at least be discussed/considered to explain the different basal conjugation rates/conjugation efficiency.

Secondly, the impact of aggregation substance in the E.coli. In the absence of any study or any result in this paper that demonstrates this relationship, one cannot speculate about the influence of aggregation substances on other species different from Enterococcus faecalis. This has not been demonstrated for other enterococcal species, and extrapolating it to other phyla is extremely speculative in the absence of scientific evidence. E. faecalis has developed other cell-to-cell strategies (e.g., QS) that could be discussed. Aggregation substance is not a virulence factor in "sensu stricto". Revise.

Finally, the results section could be organized into subheadings corresponding to the different experiments performed. This will help readers better understand the findings that align with the different objectives (analysis of the conjugation efficiency of different E. coli strains and the effect of concomitant species on the conjugation transfer )

**Editor Decision: Minor revision**

**Author's Response to Decision Letter for (FEMSMC-2024-003.r1)**

We would like to thank the reviewers for taking the time to provide constructive feedback, comments, and recommendations to our manuscript. We have addressed and incorporated the suggestions and recommendations by the reviewers, which have contributed to the improvement of the manuscript.

Below we have listed our response to the reviewers.

Reviewers' Comments to Author/s:

Reviewer: 1

Comments to the Author

I would like to thank the authors for their detailed and thorough revision of this manuscript addressing all reviewers’ comments. This has resulted in a greatly improved manuscript that highlights the key findings and novelty of this study.

There are some points still to raise however, requiring additional clarification.

Minor comments

1. Lines 328 – 329: please justify whether the potential for reduced nutrient concentration in the AUM spent media plates (0.75 x AUM) due to nutrient consumption, could have affected measured conjugation rates in Figure 2. Were additional control plates tested with 0.75 x AUM rather than 1 x AUM?

We appreciate this need for clarification from the reviewer. Indeed, we performed additional control plates with 0.75 x AUM as mentioned in the materials and methods section. These low control 0.75 x AUM nutrient conditions correspond to slightly higher conjugation rates than the high nutrient control (1x AUM) and, therefore if we compare the conjugation rates of E. coli in the presence of E. faecium and E. faecalis (as measured by conditioned medium) with this low nutrient control, these also have a relative negative effect on conjugation rate.

We had reported the 1x AUM control plates for the following reason. As it is mentioned in lines 363-364: ‘It should be noted that Gram-positive enterococci hardly grow in artificial urine medium (103-104 CFU/ml), these relatively low counts mimic their growth in urine (Flores-Mireles et al. 2015)’, our hypothesis was that the conditioned media of E. faecalis and E. faecium would therefore be closer to 1x AUM instead of 0.75 x AUM given that during its preparation for 48 hours, not much nutrient depletion is expected.

We have added in the text from lines 355 to 362: ‘We have also tested these results under additional reference conditions aimed at assessing the boundary conditions of nutrient depletion in the conditioned medium. Supplementary Figure 4 shows that under these conditions, the relative effect of enterococci on the conjugation rates of the two E. coli isolates B and F, was significant and negative in three combinations: E. coli B with E. faecalis and E. coli F with E. faecalis and E. faecium, while for E. coli B with E. faecium the effect was not significant. This indicates that primary or secondary metabolites released by E. faecium and E. faecalis are unlikely to be the leading cause of the increased conjugation rates observed in the co-culture experiments.’ Please note that in the co-culture conjugation experiments there is no nutrient depletion as they are performed in 1x AUM.

Consequently, we have created a new Supplementary Figure 4, which has been added to the supplementary material together with its caption.

2. Lines 241 and 326: please justify in the text how the selection of E. coli isolates B and F was decided for the spent media assays. Were these isolates chosen based on contrasting basal conjugation rates shown in Figure 1? How does this link with the specific isolate results described on lines 309 – 313?

We thank the reviewer for this question. We have updated the text from lines 245 to 249 to justify the choosing of E. coli isolates B and F: ‘conjugation experiments between donor strain ꞵ3914 and two E. coli isolates; B and F (Figure 1), were performed on spent media agar plates from two enterococcal isolates, and the controls on AUM agar plates. The two E. coli isolates were chosen based on contrasting conjugation rates (Figure 1)’.

It is important to mention that both isolates were chosen based on their contrasting conjugation rates shown in Figure 1, as the reviewer suggested. At the moment of performing the condition media experiments, we did not have all the results of the co-culture conjugation rate experiments. We had seen, however, the striking co-culture effect of the enterococci on the conjugation rates of E. coli. We initially chose three distinctive isolates; A, B and F, in terms of their different conjugation rates, and we tested them with enterococci spent media to see whether spent media would show similar effects. In this version we show the results for two; B and F, because we found out that the variation of A was larger than we had seen in other experiments, we decided to omit it from Figure 2 in the previous round of revision.

Regarding the specific isolate results, previously in lines 309-313 and now in lines 321-325, namely: ‘For instance, for E. coli isolate A, three UTI community members increased the conjugation rate by three orders of magnitude. For isolate C, the presence of both Enterococcus species increased the conjugation rate by two orders of magnitude. Yet, for E. coli isolate E we could not detect any significant changes to the conjugation rate due to the influence of any other of the UTI community members (Figure 1)’. Are therefore not connected to the selection of isolates but were a description of the results from the co-culture experiments and the different effects observed for each isolate.

3. Lines 328 – 330: please specify these results shown in Figure 2 more clearly, differentiating between the results found for isolates B and F. It is not immediately clear that “in three experiments the effect was not significant” relates to the total of four experiments performed over the two isolates.

We thank the reviewer for this remark. We agree that it was not immediately clear what we meant by ‘experiments’; therefore, we have reworded the paragraph to reflect that we are referring to the ‘combinations’ of the two E. coli isolates and the two enterococci. Now, from lines 349 to 352 the text reflects the following: ‘We found that the spent media experiment generally shows a rather small negative effect on the conjugation rate. Namely, in three combinations of E. coli under the influence of enterococci: E. coli B with E. faecalis and E. faecium, and E. coli F with E. faecium the effect was not significant and in one; E. coli F in the spent medium of E. faecalis, the effect was significant but negative’.

4. Lines 293 and 295: please report the full statistical results of the ANOVA tests including F statistic and degrees of freedom.

We have updated the text to include the full statistical report with F statistic and degrees of freedom. The text from lines 301 to 304 now reflect these changes: ‘We found that the effect of community members differs significantly for different E. coli isolates (two-way ANOVA, Df = 25, F value = 14.15, interaction: p < 2e-16). Moreover, we found that the basal conjugation rates, in the absence of community members, differ significantly between the six E. coli isolates (ANOVA, Df = 5, F value = 15.82, p = 6.4e-05)’.

5. Lines 298 – 299: please clarify how lack of inhibition of growth of the donor and recipient strains was inferred. Is this a qualitative assessment based on CFU/ml densities presented in Supplementary Table 2?

We thank the reviewer for pointing out this need for clarification. Indeed, we had done a qualitative assessment. We have performed an additional quantitative analysis to assess whether the changes in donors and recipients under the influence of community member species can explain the statistically significant changes in the conjugation rates presented in Figure 1. Here we recalculated the conjugation rate, for each of the E. coli isolate community member combinations, using the donor and recipient numbers from the control groups (instead of using the donors and recipient numbers from the community conjugation combinations). For instance, for isolate B in combination with E. faecium, we recalculated the conjugation efficiency by dividing the number of transconjugants (column F, cells 23-25) by the CFU/mL of donors and recipients of the control (column D and E, cells 20-22) (Supplementary Table 2). We assessed the significance of the change in conjugation rate using a one-way ANOVA and Dunnett’s post-hoc test.

We found that for E. coli isolates B and F in combination with E. faecium, changes in donor and recipient numbers in the community conjugation experiment can play a role in the increased conjugation rate observed in Figure 1. Specifically, we found that for the above-described analysis, in the case of E. coli isolates B and F with E. faecium, these interactions are not significant anymore (B with E. faecium p = 0.3941, F with E. faecium p = 0.842). Therefore, for these combinations, the changes of donors and recipients in the presence of E. faecium can partially explain the statistically significant change in conjugation rate from Figure 1 for B and F.

We have reworded the text from lines 307 to 311 to reflect the additional statistical analysis that follow in the next paragraph: ‘Assessing the effect of the bacterial interactions on the conjugation rate, we did not find that any of the other tested isolates inhibit the growth of either donor or recipient to such an extent that the variation in donors and recipients alone (compared to the control) cannot explain the increase in conjugation rate (Supplementary Table 2), except for E. coli isolates B and F in combination with E. faecium (one-way ANOVA and Dunnett’s post-hoc test, p<0.05).’

6. Lines 149 – 152: For the LB agar plates with kanamycin without DAP, please clarify why transconjugant selection was expected. Is kanamycin resistance also present and transferred on the pOXA-48 plasmid?

We appreciate this need for clarification from the reviewer. We have updated the text from lines 151 to 157 to: ‘Every donor and recipient mix, as well as the controls (donors in isolation and recipients in isolation), were plated on transconjugant-selective LB agar plates with ampicillin 100 µg/mL where only transconjugants should grow. As an additional negative control, every donor and recipient mix, as well as the controls (donors in isolation and recipients in isolation) were plated on LB agar plates with kanamycin 30 µg/mL (Sigma) without DAP, to make sure that neither donor nor recipients in isolation nor transconjugants would grow.’ We must mention that kanamycin resistance is not present in the pOXA-48 plasmid but only the donor strain E. coli ꞵ3914. We added this additional negative control because we wanted to make sure that we were not looking at contamination.

7. Line 261: include citations and references for R and the package DescTools.

Thank you for pointing out these missing references. We have updated the text in lines 267 and 268, which now state: ‘Statistical analyses were performed in R v4.1.2 (R Core Team 2021) and the package DescTools v0.99.50 (Signorell 2024)’.

8. Line 400: abbreviate the genus name for Enterococcus faecalis.

Thank you for the remark. We have updated the discussion section; therefore this change was no longer needed.

9. Line 247: capitalise the ‘E’ for Erlenmeyer.

Thank you for the remark. We have updated the text to reflect this change in line 253.

Reviewer: 2

Comments to the Author

This is my second review of the paper written by Bustamante et al. The authors analyzed the effect of concomitant bacterial taxa on the “conjugation rate”/”conjugation efficiency” of plasmids between Escherichia coli strains. The goal is to explore how often plasmid transferability is triggered by other species that coexist in polymicrobial infections, taking as a model strains from polymicrobial urinary tract infections (ITU) in the elderly. Different factors associated with plasmid acquisition, transfer, or maintenance have been extensively studied. However, the impact of concomitant species is still a neglected issue.

The authors have notably improved the original version of the manuscript by adding statistics and clarifying some technical details of the protocols. However, some reviewer´s comments on interpreting the results have not been fully addressed. Some additional comments are given for the author´s consideration.

Firstly, this referee considers it relevant to describe the lineage of the E. coli strains used. The authors believe that they may be out of the scope of this paper and might be included in future studies. I disagree with this opinion. Plasmid cost/transferability is often strain/lineage-specific; thus, the genotypic background can influence the "conjugation efficiency." Different Escherichia coli phylogroups contain different plasmid backgrounds, which could influence the uptake and transferability of incoming plasmids. Also, in the absence of any genotyping, one cannot be sure how similar these E. coli strains are (pandemic clones are widespread in both hospital and community-based settings, and they often vary in the accessory genome). I understand the authors do not wish to include more experiments, but the description of the sample seems to be relevant for interpreting the results of this work. This point should at least be discussed/considered to explain the different basal conjugation rates/conjugation efficiency.

We thank the reviewer for this important remark. In general, we definitely agree with the reviewer, however it is important to mention that without a large sample size we were hesitant to draw any firm conclusions related to the distribution of our isolates on the phylogroups in relation to their conjugation rates. Yet, we also agree that these findings could still be better embedded in academic literature, and we have added data on the corresponding sequence types and phylogroups of some of our uropathogenic E. coli isolates (based on information received from our collaborator Alan McNally, acknowledged in the acknowledgements).

We have updated part of the materials and methods section in lines 93 to 95: ‘Multi locus sequence typing, followed by phylotyping (Beghain et al. 2018) of these uropathogenic E. coli isolates was performed previously by Croxall et al. (2011a)’

We have also added text to the results section, where we relate the phylogroups to the conjugation rates from lines 334 to 341:

‘We find that the conjugation rate levels for the tested isolates do not correspond to their assigned phylogroups (Croxall et al. 2011a) (Supplementary Table 4). For instance, isolates A and D are both part of ECOR group B2 (Ochman and Selander 1984) as well as two of the isolates that didn’t take up the plasmid (from the conjugation rates of pOXA-48 to uropathogenic E. coli on LB experiment). One of the non-conjugative B2 isolates belongs to the sequence type 131, a drug-resistant uropathogenic strain of E. coli widely disseminated among both community and hospital patients (Lau et al. 2008). This suggests that strain specific genetic components rather than phylogroup classification are important for the ability to take up the pOXA-48 plasmid’.

Furthermore, because we fully agree that genetic background effects are important and relevant for clinical understanding, we have further updated the discussion section from lines 393 to 413 to provide details at the general level on the effects of genotype and conjugation efficiencies, emphasizing that phylogroup classification does not correspond to similar conjugation rates:

‘Nine uropathogenic E. coli isolates were initially selected for conjugation with pOXA-48. Only six of these E. coli isolates took up the plasmid, as verified by PCR with pOXA-48 specific primers (Materials and Methods). The fact that each of them was associated with unique basal conjugation rates indicates that there are host-dependent genetic background interactions that determine these rates, which is in accordance with other findings (Alonso-del Valle et al. 2023; Benz and Hall 2023). Regarding the different sequence types and phylogroups assigned to the tested E. coli isolates, here we find that the levels of conjugation rates, and even the ability to take up pOXA-48 plasmid, were not consistent with those. These results contrast previous literature showing that specific phylogroups are associated with specific transferability of plasmids (Carattoli 2011) that are associated with particular pathogenic phenotypes or multidrug resistance, as is the case with sequence type 131 (Lau et al. 2008).

In general, the presence of a plasmid in a new bacterial host might come with fitness costs, for which single compensatory mutations are often sufficient to completely ameliorate such costs, suggesting that these are caused by specific genetic conflicts rather than generic properties of plasmids, such as their size, metabolic burden, or gene expression level (Hall et al. 2021). For instance, plasmid-encoded extended-spectrum beta-lactamase gene acquisition in diverse E. coli lineages can drive strain-specific interactions, such as chromosomal mutations affecting metabolic and regulatory functions that ultimately might affect plasmid stability conjugation efficiency (Carrilero et al. 2023). In the wider context of bacterial communities, both the cost of plasmid carriage and its long-term maintenance in a focal strain are found to depend on the presence of competitors, and these interactions are species-speciﬁc too (Sünderhauf et al. 2023).’

Secondly, the impact of aggregation substance in the E. coli. In the absence of any study or any result in this paper that demonstrates this relationship, one cannot speculate about the influence of aggregation substances on other species different from Enterococcus faecalis. This has not been demonstrated for other enterococcal species and extrapolating it to other phyla is extremely speculative in the absence of scientific evidence. E. faecalis has developed other cell-to-cell strategies (e.g., QS) that could be discussed. Aggregation substance is not a virulence factor in "sensu stricto". Revise.

Thank you for pointing out this need for clarification. We have updated the text in the discussion section, where in lines 435-452 it is now stated: ‘Proximity between cells, such as found in bioﬁlms, is known to facilitate the spread of antibiotic resistance by promoting horizontal gene transfer (Fux et al. 2005) e.g. via quorum sensing, which leads to changes in gene expression, potentially expediting the acquisition of antibiotic resistance (Schroeder, Brooks and Brooks 2017; Lin et al. 2021). However, if the increased conjugation rates would be caused by quorum sensing molecules released by enterococci, then we expected to observe an increase in conjugation rates in the presence of conditioned medium prepared from enterococci, and that was not the case. Our findings therefore suggest that the nature of the co-culture and its direct interactions are important for these increased conjugation rates in artificial urine medium. Specifically, we hypothesize that physical contact or proximity (Stalder and Top 2016), between these species may play a role. It is worth mentioning that pOXA-48 is an Inc L/M plasmid type that encodes short, rigid pili known to have higher transfer frequencies on solid surfaces compared to liquid (Bradley 1980). Various types of cell-to-cell contact have been shown to be involved in promoting the transfer of genetic material within species (Morawska and Kuipers 2022). One alternative hypothesis would be that the presence of some Gram-positive species is strengthening the interaction between the two E. coli strains (donor and recipient) as a sort of defense mechanism, limiting the direct interaction of the Gram-positive ‘intruder’ with the interacting E. coli strains. This is reminiscent of biofilm formation as a defense mechanism (Donlan and Costerton 2002; Kumar et al. 2017).’

Finally, the results section could be organized into subheadings corresponding to the different experiments performed. This will help readers better understand the findings that align with the different objectives (analysis of the conjugation efficiency of different E. coli strains and the effect of concomitant species on the conjugation transfer).

We thank the reviewer for this suggestion. We have incorporated it, and the results section has been organized to reflect the different experiments that were performed. Specifically, line 284 now states: ‘Conjugation rates of pOXA-48 to uropathogenic E. coli in LB’; lines 291 to 292 now have the following text: ‘Conjugation rates of pOXA-48 to uropathogenic E. coli with and without community members on AUM’; and in line 342, the text is: ‘Conjugation rates of pOXA-48 to uropathogenic E. coli in artificial urine spent media’.

**Reviews of manuscript FEMSMC-2024-003.r2**

**Reviewer 1: Sarah Duxbury, University of Warwick**

**Recommendation: Minor Revision**

I would like to thank the authors again for their detailed revision of the manuscript, addressing reviewer comments and the inclusion of additional data, which have improved the manuscript. Comments have now been addressed except a few minor inconsistencies in wording requiring editing to avoid misinterpretation of the results.

Minor comments

1. The authors have clarified the difference in their definitions of conjugation efficiency and rate on lines 85 – 88, however this terminology is not kept consistent with the experiments described throughout the manuscript. Please check through the manuscript and adjust terminology appropriately, including on lines 122-124, 284, 294, 296, 337, 389-390, 454, 471.

2. Lines 307 – 311: Clarification on wording is needed, including simplification of a very lengthy sentence that is difficult to interpret. Please make these statements more explicit, including details given in the response to point 5 of Reviewer 1 in the previous manuscript revision.

3. Line 317: Please correct the result description, as *P. mirabilis* alters the conjugation rates in **three** of the six *E. coli* species, to reflect the results shown in Figure 1.

**Reviewer 2**

**Recommendation: Accept**

The authors have appropriately addressed all the suggested changes, and this referee has no further comments or suggestions for this interesting work.

**Editor Decision: Minor revision**

**Reviews of manuscript FEMSMC-2024-003.r3**

**Editor Decision: Accept**
